# Supplementary material for: Linking electrocatalytic turnover to elementary step rates in hydrocarbon fuel oxidation
Source: Nat Commun. 2025 Oct 14;16:9100. doi: 10.1038/s41467-025-63910-4 (PMC12521664; doi:10.1038/s41467-025-63910-4)
Supplement: Supplementary file 1 — Supplementary Information [file 41467_2025_63910_MOESM1_ESM.pdf]

Supplementary Information

Linking Electrocatalytic Turnover to Elementary Step Rates in Hydrocarbon Fuel Oxidation

*Alexander J. Zielinski [1], Christine Lucky [2], and Marcel Schreier [1,2]**

[1] Department of Chemistry, University of Wisconsin–Madison, Madison, Wisconsin 53706, United States

[2] Department of Chemical and Biological Engineering, University of Wisconsin–Madison, Madison, Wisconsin 53706, United States

AUTHOR INFORMATION

Corresponding Author

* Prof. Marcel Schreier

Department of Chemical and Biological Engineering & Department of Chemistry

University of Wisconsin–Madison

1415 Engineering Drive, Madison, WI 53706

E-mail: mschreier2@wisc.edu

Table of Contents

- **Experimental Details**
  - I. Materials and Methods
  - II. Platinized Pt Catalyst Preparation
  - III. Cyclic Voltammetric Study of Propane Oxidation Details
  - IV. Electrochemical Mass Spectrometry Experimental Setup
  - V. Calibration of Electrochemical Mass Spectrometer
- **Data Collection and Analysis of Electrochemical Mass Spectrometry Studies**
  - I. General Procedures
  - II. Constant Potential Oxidation of Propane
  - III. Rate of Propane Adsorption
  - IV. Adsorbate Conversion Above 0.5 V
  - V. Adsorbate Conversion Below 0.5 V
  - VI. Oxidation of CO
  - VII. Enhancement of Propane Oxidation Rates via Pulsed Potential Sequences
- **Comments on the Interpretation of Experimental Results**
- **Total Oxidation of Propane Chemical Equation**
- **Supporting Data and Data Fitting**
- **Additional Data**
- **EC-MS Calibration Curves and Data Normalization**
- **He Control Experiments**
- **Propane to He Gas Exchange Control Experiments**
- **Broad Spectrum Product Detection**
- **Estimation of Diffusion-limited Adsorption Rate**
- **Additional data – Cyclic Voltammetric Study of Propane Oxidation at 80 °C**
- **Photographs of Experimental Setups**
  - I. EC-MS Setup
  - II. Rotating Disc Electrode Setup
- **Catalyst Characterization**
  - I. Catalyst Preparation for Material Characterization
  - II. X-ray Diffraction
  - III. X-ray Photoelectron Spectroscopy
  - IV. Scanning Electron Microscopy
- **EC-MS Electrochemical Cell Configuration**
- **References**

Experimental details

I. Materials and Methods

All glassware and polytetrafluoroethylene (PTFE) electrochemical cell components used in the preparation of electrolytes and during experiments were cleaned using piranha solution formulated from 85% H_2SO_4 (Sigma-Aldrich, ACS Reagent grade, 95.0-98.0%) and 15% H_2O_2 (Honeywell, semiconductor grade, 30% or Fisher, ACS grade, 30%) then rinsed thoroughly with DI water (ACS Reagent Grade, ASTM Type 1, LabChem). Glass electrochemical cell components were dried in an oven at 80 °C and then cooled to room temperature under ambient conditions before use. PTFE electrochemical cell components were dried with compressed air before use.

Perchloric acid (70%, 99.999% trace metals basis) was purchased from Millipore-Sigma. 1 M perchloric acid was used as the electrolyte for all reported experiments. This solution was prepared by diluting 70% perchloric acid with purified water (18.2 MΩ, Millipore Milli-Q Reference A+, TOC = 2 ppb). Adjustable micropipettes (Eppendorf, Research Plus) and volumetric glassware were used to prepare the diluted perchloric acid. 500 mL of solution was prepared and stored in a 500 mL glass bottle (piranha solution cleaned) with polypropylene cap (VWR). Before use, approximately 10 mL of 1 M perchloric acid was transferred to a 50 mL sterile centrifuge tube (Falcon, Corning). This solution was then sparged with He for at least 20 min using a glass gas-dispersion tube (piranha solution cleaned) to removed dissolved gas. Three batches of electrolyte were used during this work and were all prepared and stored in the same way. The first batch was prepared on 10/29/2023 and had a pH value of 0.15 ± 0.01, the second batch was prepared on 1/3/2024 and had a pH value of 0.16 ± 0.02, and the third batch was prepared on 4/2/2024 and had a pH value of 0.15 ± 0.06. Measurements of pH were performed using a Mettler-Toledo FiveGo F2 with LE438 probe.

Helium gas (Ultra High Purity Grade), Propane (Research Grade), H_2 (Ultra High Purity 5.0 Grade), CO_2 (Research Grade), and CO (Research Plus Grade) were purchased from Airgas.

II. Platinized Pt Catalyst Preparation

A 5 mm diameter platinum disk electrode (99.995%, Pine Research) was polished using progressively finer alumina slurries (1.0, 0.3 and 0.05 µm, Allied High Tech) and sonicated (Braunsen 3800) in DI water (ACS Reagent Grade, ASTM Type 1, LabChem) after each polishing step. High surface area platinized platinum was then deposited via electrodeposition from a solution of 0.072 M H_2PtCl_6 (99.9% trace metal basis, Millipore-Sigma) and 1.3×10^{-4} M $Pb(C_2H_3O_2)_2$ (99.999% trace metal basis, Millipore-Sigma) by applying -30 mA cm^{-2} (-5.89 mA ÷ 0.196 cm^2_{geo}) for 500 s.[1,2] The application of current was controlled by a Biologic SP-200 potentiostat with EC-lab (Version 11.33) software. Catalyst deposition was performed in a two-electrode configuration with a Pt wire (Kurt J. Lesker, 99.99%), flame cleaned with a butane torch, used as the counter electrode. Electrodeposition was performed at room temperature. After deposition, the catalyst was submerged in DI water (ACS Reagent Grade, ASTM Type 1, LabChem) several times to rinse off residual platinizing solution. The catalyst loading was approximated to be 2.19 mg cm^{-2} by recording the mass (Mettler-Toledo, XSR205) difference of the 0.196 cm^2_{geo} surface area electrode before and after platinizing, rinsing, and drying the electrode. Small particles of deposited Pt were often dislodged during the rinsing step which affects catalyst loading for each electrode preparation. To account for this and drift in the electroactive

surface area during successive electrode use, all voltammetric current and ionic current signals were normalized by dividing the signals by the absolute value of the charge required to reduce the platinum oxide (C_{o-r}) formed during the second CV cycle performed between 0 – 1.4 V at 50 mV s^{-1} after experiment completion.[3] An example CV with charge integration is shown in Supplementary Fig. 14. Representative electrochemical surface area (ECSA) measurements were performed and approximated to be 108 ± 4 cm^2 by analyzing the oxidative charge associated with the desorption of hydrogen in the hydrogen underpotential deposition (H_{UPD}) region calculated using a factor of 210 μC cm^{-2} (Supplementary Fig. 35a).[4] This ECSA value agrees with 100 ± 8 cm^2 estimated by analyzing the He background subtracted charge passed during the oxidation of a monolayer of CO adsorbed to the electrode at 0.1 V for 20 min and calculated using a factor of 420 μC cm^{-2} (Supplementary Fig. 35b).[4] These ECSA measurements are associated with a C_{o-r} value of –0.0278 ± 0.001 C (Supplementary Fig. 35a).

Each electrode was pre-conditioned using 10 cycles of cyclic voltammetry (CV) at 50 mV s^{-1} from 0 to 1.4 V vs SHE in He-saturated 1 M perchloric acid at room temperature. An additional 10 CV cycles in He-saturated electrolyte at the reaction temperature and 20 cycles in substrate-saturated electrolyte at the reaction temperature were performed before performing experiments. A typical CV for the EC-MS set-up is shown in Supplementary Fig. 9. Catalyst characterization was performed using X-ray diffraction (XRD), X-ray photoelectron spectroscopy (XPS), and scanning electron microscopy (SEM).

III. Cyclic Voltammetric Study of Propane Oxidation Details

Cyclic voltammetry experiments were performed in the EC-MS cell. After electrolyte was injected into the cell, 10 CV cycles at 50 mV s^{-1} from 0.1 – 1.3 V was performed to pre-condition the electrode and electrolyte. Before each experiment, a pulse potential cleaning procedure (Supplementary Fig. 1) was performed to ensure no propane that may have adsorbed to the catalyst surface between experiments was included in the measurement. Following cleaning, the electrode potential procedure shown in Fig. 2a was performed. After adsorbing propane for 60, 300, or 900 s at 0.3 V, CVs were carried out between 0.3 V and 1.3 V at 50 mV s^{-1} and are plotted in Fig. 2b. A second CV cycle was performed immediately following the first cycle where Cycle 2 from the 900 s propane adsorption run was included in Fig. 2b. He blank control experiments were performed with adsorption for 900 s at 0.3 V.

IV. Electrochemical Mass Spectrometry Experimental Setup

A Spectro Inlets (Denmark) Electrochemical Mass Spectrometer controlled by Zilien software (Spectro Inlets, Denmark, Version 2.5.0) was used to perform all EC-MS measurements.[5] All EC-MS experiments were conducted in a PTFE stagnant thin-layer electrochemical cell (Supplementary Fig. 36) which was interfaced with the gas-permeable microporous membrane MS inlet chip (aqueous chip, Spectro Inlets) as shown in Supplementary Figs. 36, 37, 38, and 39. Electrolyte was injected into the electrochemical cell using single-use Henke-Ject syringes (HENKE SASS WOLF, Luer Lock, sterile, 3 mL, 4020-X00V0). The approximate volumes of electrolyte in the working, counter, and reference electrode compartments were 9.5 μL, 1 mL, and 2 mL, respectively. The cell compartments were not separated by frits or membranes. The electrochemical cell and cell mounting block was heated to 60 ± 1 °C for all experiments unless otherwise stated using heating tape (BriskHeat), controlled by a BriskHeat controller (SDXJA),

monitored using a thermocouple (HH802U, OMEGA Engineering) positioned between the electrochemical cell and the EC-MS cell mounting block, and insulated with glass wool.

A coiled Pt wire (Kurt J. Lesker, 99.99%), flame cleaned with a butane torch, and a Ag/AgCl electrode (BASi, RE-5B, 3.0 M KCl) were used as the counter and reference electrodes, respectively. Reference electrodes were calibrated by measuring the potential difference between an identical reference electrode which was never used in experiments and assumed to have a value of 0.210 V vs SHE.

When using propane as the substrate, CO_2 signals were quantified from the m/z 16 signal due to large overlap between CO_2 and propane m/z 44 MS signals. MS signals were not processed using a deconvolution protocol. MS ionic current signals and peaks were integrated in reference to the signal baseline measured after experiment completion using Origin Lab (Version 2022b). All gases were supplied via the Spectro Inlets EC-MS gas manifold mass flow controllers and introduced to the electrolyte through the gas-permeable membrane chip. During experiments that involved mid-experiment gas switching (Oxidation of *CO, Fig. 6), gases were initially flown at 10 sccm to facilitate rapid removal of the previously supplied gas from the electrolyte, then flown at 1 sccm during the last 1 min of a gas exchange step. All gases were flown at 1 sccm during all other processes.

Applied potentials were controlled by a Biologic SP-200 potentiostat with EC-lab (Version 11.33) software interfaced with the Zilien (Version 2.5.0) EC-MS software. Either a 10 Ω, 30 Ω, or 100 Ω resistor was connected in series with the working electrode to improve potentiostat signal stability. Potentiostatic electrochemical impedance spectroscopy from 200 to 200,000 Hz with an amplitude of 10 mV was performed before each EC-MS experiment. Cell resistance is reported for relevant figures and tables. Due to low faradaic currents, iR values were considered negligible and data was not iR corrected.

V. Calibration of Electrochemical Mass Spectrometer

The EC-MS system was calibrated so that ionic currents could be converted to analyte flux. First, a calibration factor to calculate analyte flux from analyte concentration was determined.[5] This was accomplished by performing both internal and external calibrations for H_2 gas. Internal calibration of H_2 was completed by measuring the m/z 2 ionic current while producing H_2 via the hydrogen evolution reaction (HER) in 1 M perchloric acid. A two-electrode setup with a polished Pt disk (99.995%, Pine Research) and a Pt wire (99.99%, Kurt J. Lesker) were used as the working and counter electrodes, respectively. Applied currents were controlled by a Biologic SP-200 potentiostat with EC-lab (Version 11.33) software interfaced with the Zilien EC-MS software. Assuming 100% Faradaic efficiency for H_2 production, constant-current experiments from −1 to −10 μA were performed to generate H_2 at a known rate (nmol s^{-1}) and the corresponding m/z 2 ionic currents (A) were measured (Supplementary Fig. 13a). The employed EC-MS system is reported to have a 100% collection efficiency, therefore under steady-state H_2 production, the H_2 production rate is expected to be equivalent to the H_2 flux (nmol s^{-1}) reaching the MS for detection. Leveraging this 1:1 relationship, a calibration curve relating the m/z 2 ionic current to the H_2 flux was constructed (Supplementary Fig. 13b).

An external H_2 calibration was then performed where the m/z 2 ionic current was measured when varying concentrations of H_2 were introduced to the EC-MS system while the electrochemical cell was mounted and filled with Milli-Q water. This was accomplished by using H_2, diluted in He from 5,000 to 25,000 ppm, as the carrier gas. The m/z 2 ionic current vs H_2 concentration data was plotted in Supplementary Fig. 13c.

Next, the data from both the internal and external H_2 calibrations were used to relate concentration to flux. Assuming that the flux of He is constant for dilute mixtures, the total flux of gas to the MS vacuum chamber was calculated as,

$$Total\ Gas\ Flux = \frac{\left(S_{x_{H_2}}^{m/z\,2} - b\right)/m}{x_{H_2}}, \tag{S1}$$

where $S_{x_{H_2}}^{m/z\,2}$ is the m/z 2 MS ionic current signal obtained during the hydrogen external calibration for the hydrogen concentration x_{H_2}, b is the intercept from the H_2 internal calibration (m/z 2 ionic current background), m is the slope of the H_2 internal calibration, and x_{H_2} is the mole fraction of H_2 from the external calibration. Using Supplementary Eq. 1, the total flux of gas reaching the MS was calculated for each concentration of H_2 introduced during the external calibration. An average value of 7.62 nmol s^{-1} was determined. This value was used for the conversion of concentration to flux for other externally calibrated gases.

To quantify CO_2 produced during EC-MS experiments, an external calibration was performed by introducing CO_2, diluted between 400 and 250,000 ppm with He, to the EC-MS system. The mole fraction of CO_2 introduced to the system was then multiplied by 7.62 nmol s^{-1} to obtain the flux of CO_2 (Supplementary Fig. 14a). This conversion allowed a calibration curve of m/z 16 ionic current vs CO_2 flux to be constructed (Supplementary Fig. 14b). Performing a linear fit of this data yielded a calibration factor of 5.91×10^{-10} A s $nmol^{-1}$. This calibration factor was used to convert the m/z 16 ionic current measured during each experiment to the flux of CO_2.

During calibration, the m/z 4 ionic current, while delivering 1 sccm He carrier gas to the EC-MS, was recorded. For every set of experiments, the m/z 4 ionic current, while delivering 1 sccm He carrier gas to the EC-MS, was also recorded and referenced to the measurement recorded during calibration. A He carrier gas chip correction factor was then implemented to accounts for slight variability in the MS tuning, secondary electron multiplier signal enhancement, atmospheric pressure, and membrane chip capillary volume. The He carrier gas chip correction factor was calculated as,

$$\text{He chip correction factor} = \frac{m/z\ 4\ ionic\ current\ (calibration)}{m/z\ 4\ ionic\ current\ (experiment)}. \tag{S2}$$

This correction factor was determined for every set of experiments. The calibration factor for CO_2 was multiplied by the determined He carrier gas chip correction factor before using it to convert m/z 16 ionic current to CO_2 flux. This correction was used in previous publications from our group and is similar to that performed by others.[2,5–7] All EC-MS calibration experiments were performed at room temperature.

The detection of oxygenates using the EC-MS system was evaluated by recording the ionic current for increasing concentration of methanol (Millipore, LiChrosolv, 99.9%), ethanol (200 proof, Sigma-Aldrich), and 2-propanol (ACS reagent, ≥99.5%, Sigma-Aldrich) prepared using Milli-Q water. The cell was assembled with a polished Pt stub (99.995%, Pine Research) in the working electrode position while no counter or reference electrode was used. 1 sccm He was flowed during these measurements. These experiments were performed at room temperature. The results are shown in Supplementary Fig. 16. No correlation between m/z 16 and alcohol concentration was observed.

Data Collection and Analysis of Electrochemical Mass Spectrometry Studies

I. General Procedures

Except where noted, all experiments were carried out using platinized Pt catalysts and 1 M perchloric acid electrolyte at 60 ± 1 °C. Unless otherwise stated, all experiments were performed in a propane saturated electrolyte with a propane flow rate of 1 sccm. CO_2 yields were quantified from the m/z 16 MS signals.

II. Constant Potential Oxidation of Propane

The steady-state constant-potential oxidation of propane (turnover) on Pt catalysts was studied as a function of the applied electrode potential by measuring CO_2 production. As such, we used EC-MS to quantify the amount of CO_2 produced at potentials between 0.4 and 1.1 V (Fig. 3a). Each experiment was preceded by an electrode cleaning step at 1.4 V, followed by 0.05 V for 20 s each, repeated 3 times (Supplementary Fig. 1). To measure the rate of propane oxidation, without convolution by pre-adsorbed propane, we applied the potential $E_{turnover}$ after biasing the electrode at 0.05 V for 180 s, which inhibited propane adsorption. After a time $t_{turnover}$, we changed the potential to 0.3 V, which arrested oxidation and allowed the m/z 16 signal to decay to the baseline. The m/z 16 ionic current during $E_{turnover}$ and the following 0.3 V stabilization period was integrated in 60 s segments to trace CO_2 production over time. Cumulative CO_2 yields were calculated from these integrals and used to calculate the consumption of propane according to the stoichiometric coefficient of 3, according to Supplementary Eq. 3. The consumption of propane was plotted in Fig. 3b. The rate of continuous propane oxidation was determined by fitted linear curves to the cumulative CO_2 yield data and are shown in Supplementary Table 1 where the slope is the continuous CO_2 production rate. The rate of propane turnover is calculated by dividing the CO_2 production rate by the stoichiometric coefficient of 3, according to Supplementary Eq. 3, and is plotted in Fig. 3c.

III. Rate of Propane Adsorption

The adsorption of propane was studied using the oxidative stripping sequence shown in Fig. 4a. Each experiment was preceded by an electrode cleaning step at 1.4 V, followed by 0.05 V for 20 s each, repeated 3 times (Supplementary Fig. 1). After cleaning, the MS signal was allowed to stabilize for 160 s at 0.05 V. The potential was then stepped to the potential E_{ads} for the duration t_{ads}. Upon completion of the adsorption step, an oxidative stripping potential of 1.3 V was applied to rapidly oxidize all accumulated propane derived intermediates to CO_2 and prevent further adsorption of propane due to the formation of a passivating oxide film. The CO_2 yield was calculated by integrating the m/z 16 ionic current during E_{ads} and the 1.3 V stripping step. Blank experiments were performed with $t_{ads} = 0$ s and the CO_2 yields from these experiments were averaged and subtracted from the data.

In this study, EC-MS allows us to monitor CO_2 production during both the adsorption and oxidation steps. This differs from previous studies which generally only considered contributions during the oxidation step. As shown in the Figs. 3b and 3c, adsorption potentials between 0.5 – 0.9 V will facilitate oxidative turnover and produce CO_2. Here, we advocate that CO_2 produced due to turnover during the adsorption step must be included in the total CO_2 yield when calculating the propane adsorption rate. This interpretation is rationalized as the CO_2 produced due to turnover must have come from propane that was adsorbed to the surface during the adsorption

step. Thus, measuring just the CO_2 yield upon application of the oxidation step (as has been done in previous studies) only provides the quantity of accumulated adsorbates and not the total of species that have undergo adsorption.

CO_2 yield from these adsorption experiments were used to calculate the quantity of adsorbed propane, by dividing the CO_2 yields by the stoichiometric coefficient of 3, according to Supplementary Eq. 3. Dividing these CO_2 yields by the corresponding adsorption time gives the rate of propane adsorption in terms of CO_2 and are plotted in Supplementary Fig. 4. Finally, these rate values were divided by the stoichiometric coefficient of 3, according to Supplementary Eq. 3, providing the rate of propane adsorption in terms of propane. The rate of propane adsorption calculated from 30 s adsorption time data is plotted in Fig. 4c.

Three CV cycles from 0 to 1.4 V at 50 mV s^{-1} were performed at the end of each data set collection and the charge passed during the Pt oxide reduction peak on the second cycle was used to normalize for the surface area of the electrode. Experiments were repeated from entirely new sample preparation, fresh electrolyte, and freshly cleaned cell setup at least two times. The data shown in Fig. 4b corresponds to the average of these repeats and error bars show the standard deviation. The averaged data was used to calculate the adsorption rates.

Propane adsorption (Fig. 4b) over time was observed to have a slight non-linear decrease with greater deviations from non-linearity occurring at longer adsorption times. These non-linear trends prevent us from performing linear fits to determine propane adsorption rates. Instead, CO_2 yields were divided by the adsorption time which allowed the slower rate of adsorption at longer adsorption time to be represented. The adsorption rate calculated using different adsorption time points are presented in Supplementary Fig. 4. It was observed that adsorption rate generally decreases with increased adsorption time and supports the hypothesis that adsorbate accumulation hinders further adsorption of propane.

IV. Adsorbate Conversion Above 0.5 V

<u>1. Measuring the total CO_2 yield from adsorbate conversion and constant-potential oxidation during the application of conversion potentials</u>

The oxidation of propane derived CO intermediates to CO_2 and the conversion of propane derived multi-carbon intermediates to CO_2 was studied using the potential program shown in Fig. 5a. Each experiment was preceded by an electrode cleaning step at 1.4 V, followed by 0.05 V for 20 s each, repeated 3 times (Supplementary Fig. 1). After cleaning, the MS signal was allowed to stabilize for 120 s at 0.05 V. Then, propane was pre-adsorbed at 0.3 V for 120 s. This procedure forms a reproducible quantity of *CO and multi-carbon adsorbates on the catalyst surface before each conversion step. After adsorption, the potential was then increased to a more oxidative potential (E_{conv}) for the desired conversion time (t_{conv}). The potential was then reduced back to the adsorption potential (0.3 V) for 3 min to prevent further CO_2 production and to allow the CO_2 flux to stabilize. A 1.4 V oxidative step was then performed to clean the catalyst surface. The m/z 16 MS signal during E_{conv} and the 0.3 V stabilization period was integrated to determine the total CO_2 yield (Supplementary Fig. 5a).

Three CV cycles from 0 to 1.4 V at 50 mV s^{-1} were performed after each experiment and the charge passed during the Pt oxide reduction peak on the second cycle was used to normalize for

the surface area of the electrode. Each experiment was also followed by its matching turnover experiment discussed below. Experiments were repeated from entirely new sample preparation, fresh electrolyte, and freshly cleaned cell setup at least two times. The data shown in Supplementary Fig. 5a corresponds to the average of at least two repeats and error bars show the standard deviation. The averaged data was used to calculate the conversion rates.

In these experiments, the total CO_2 yield has three CO_2 sources. These sources include the oxidation of pre-adsorbed *CO, the oxidation of pre-adsorbed multi-carbon species, and the constant-potential oxidation of non-preabsorbed propane (turnover). To determine the rate at which multi-carbon adsorbates are converted to *CO, we isolated the CO_2 yield from just the multi-carbon adsorbates by preforming additional experiments and correcting for the total CO_2 yields for the CO_2 yields attributed to pre-adsorbed *CO oxidation and turnover.

2. Correction for CO_2 generated due to steady-state constant-potential oxidation of propane (turnover)

To correct for CO_2 attributed to turnover, an additional experiment was performed directly following each experiment for a given E_{conv} and t_{conv} condition. This follow-up experiment measured the steady-state constant-potential oxidation of propane (turnover) as a function of the applied electrode potential by measuring the CO_2 production. The electrode potential program shown in Fig. 3a was used and each experiment was preceded by an electrode cleaning step at 1.4 V, followed by 0.05 V for 20 s each, repeated 3 times (Supplementary Fig. 1). To measure the rate of propane oxidation, without convolution by pre-adsorbed propane, we applied the potential $E_{turnover}$ after biasing the electrode at 0.05 V for 120 s, which inhibited propane adsorption. After a time $t_{turnover}$, we changed the potential to 0.3 V, which arrested oxidation and allowed the m/z 16 signal to decay to the baseline. Each of these experiments was performed where $E_{turonver} = E_{conv}$ and $t_{turonver} = t_{conv}$ from the preceding (Fig. 5a) experiment. The m/z 16 ionic current during $E_{turnover}$ and the following 0.3 V stabilization period was integrated to determine the CO_2 yield due to turnover. Each CO_2 yield attributed to turnover is shown in Fig. 5b and Supplementary Fig. 5b and was subtracted from the total CO_2 yield value obtained from its preceding experiment (Supplementary Fig. 5a) to give the turnover corrected CO_2 yield. The turnover corrected CO_2 yield was divided by the stoichiometric coefficient of 3, according to Supplementary Eq. 3, yielding the quantity of propane conversion, which was plotted in Fig. 5c.

Three CV cycles from 0 to 1.4 V at 50 mV s^{-1} were performed after each experiment and the charge passed during the Pt oxide reduction peak on the second cycle was used to normalize for the surface area of the electrode. Experiments were repeated from entirely new sample preparation, fresh electrolyte, and freshly cleaned cell setup at least two times. The data shown in Fig. 5b and Supplementary Fig. 5b corresponds to the average of at least two repeats and error bars show the standard deviation. The averaged data was used to calculate the conversion rates.

3. Correction for the oxidation of pre-adsorbed CO

To estimate the CO_2 yield attributed to just pre-adsorbed *CO oxidation, the electrochemical program shown in Fig. 5e with E_{conv} = 0.3 V and t_{conv} = 120 s was performed periodically during a data set. Each experiment was preceded by an electrode cleaning step at 1.4 V, followed by 0.05 V for 20 s each, repeated 3 times (Supplementary Fig. 1). After cleaning, the MS signal was allowed to stabilize for 120 s at 0.05 V. Then, propane was pre-adsorbed at 0.3 V for 120 s. This procedure

forms a reproducible quantity of CO and multi-carbon adsorbates on the catalyst surface before each oxidation step. After adsorption, linear sweep voltammetry (LSV) was then performed from 0.3 V to 0.875 V at a scan rate of 100 mV s^{-1}. Upon reaching 0.875 V, the potential was stepped to 0.3 V to prevent further CO_2 production. The peak (corresponding to Peak I in Fig. 2b and attributed to the oxidation of *CO) of the LSV was integrated to determine the charge passed during the oxidation of the *CO intermediate to CO_2 (Supplementary Fig. 6). The quantity of CO_2 produced was then calculated from this charge assuming 100% faradaic efficiency for the oxidation of *CO to CO_2. This CO_2 yield was corrected for additional propane adsorption, conversion, and turnover that occurs during the LSV by performing a control experiment with zero pre-adsorption. The resulting control experiment LSV *CO oxidation peak was analyzed as described above and the CO_2 yield subtracted to give the CO_2 yield attributed to the oxidation of pre-adsorbed *CO. The CO_2 yield attributed to the oxidation of pre-adsorbed *CO collected throughout a single data set were averaged. This averaged *CO value was then subtracted from the turnover corrected CO_2 yields for that data set and the CO_2 yields attributed exclusively to multi-carbon adsorbate conversion were plotted in Supplementary Fig. 5c. Negative CO_2 yield values in Supplementary Fig. 5c indicate that the oxidation of pre-adsorbed CO is the primary CO_2 source for that data point. Positive CO_2 yield values in Supplementary Fig. 5c indicate that the conversion and oxidation of multi-carbon adsorbates is the primary source of CO_2. Three CV cycles from 0 to 1.4 V at 50 mV s^{-1} were performed after each experiment and the charge passed during the Pt oxide reduction peak on the second cycle was used to normalize for the surface area of the electrode.

The quantity of pre-adsorbed *CO formed during all Adsorbate Conversion Above 0.5 V data sets were averaged (Supplementary Table 18b). This average value was added as a blue dashed line in Fig. 5c to give the reader an approximation as to the contribution that pre-adsorbed *CO has on the conversion process.

4. Determination of propane adsorbate conversion rates above 0.5 V

The rate of conversion of multi-carbon adsorbates was calculated by fitting the initial linear segments of positive CO_2 yield data as shown in Supplementary Fig. 5c. These fits are shown in Supplementary Table 2 where the slope represents the conversion rate in terms of CO_2. Finally, this rate was divided by the stoichiometric coefficient of 3, according to Supplementary Eq. 3, providing the rate at which multi-carbon adsorbates are converted to *CO in terms of propane which was plotted in Fig. 5d.

V. Adsorbate Conversion Below 0.5 V

At potentials below 0.5 V, *CO accumulates on the surface instead of being rapidly oxidized to CO_2. Given that *CO is generated by the conversion of multi-carbon adsorbates, we can measure the rate of conversion below 0.5 V by quantifying the amount of *CO that accumulates as a function of potential and time as shown in Fig. 5e. It is noted that while adsorption and conversion are occurring concurrently in this experiment, the CV experiments in Fig. 2 suggest that conversion is the slower of the two reactions as evidenced by the formation of multiple adsorbate oxidation peaks (if conversion was faster, we would expect only Peak I to be observed).

Each experiment shown in Fig. 5e was preceded by an electrode cleaning step at 1.4 V, followed by 0.05 V for 20 sec each, repeated 3 times (Supplementary Fig. 1). After cleaning, the MS signal

was allowed to stabilize for 120 s at 0.05 V. Then, propane was pre-adsorbed at E_{conv} for t_{conv}. This procedure forms *CO and multi-carbon adsorbates on the catalyst surface. After adsorption, linear sweep voltammetry (LSV) was then performed from E_{conv} to 0.875 V at a scan rate of 100 mV s^{-1}. Upon reaching 0.875 V, the potential was stepped to 0.3 V to prevent further CO_2 production. The peak (corresponding to Peak I in Fig. 2b and attributed to the oxidation of *CO) of the LSV was integrated to determine the charge passed during the oxidation of the *CO intermediate to CO_2 (Supplementary Fig. 6). The quantity of CO_2 produced was then calculated from this charge assuming a 2 e^- oxidation per *CO molecule for the oxidation of *CO to CO_2. This CO_2 yield was corrected for additional propane adsorption, conversion, and turnover that occurs during the LSV by performing a control experiment where t_{conv} = 0 s. The resulting control experiment LSV *CO oxidation peak was analyzed as described above and the CO_2 yield subtracted to give the CO_2 yield attributed to the oxidation of pre-adsorbed *CO formed as the result of conversion. The CO_2 yields are listed in Supplementary Table 19 and plotted in Supplementary Fig. 11. The CO_2 yield was divided by the stoichiometric coefficient of 3, according to Supplementary Eq. 3, providing the quantity of converted propane and was plotted in Fig. 5f. Dividing the CO_2 yields by the conversion time gave the rate of propane conversion in terms of CO_2 production. Finally, this rate was divided by the stoichiometric coefficient of 3, according to Supplementary Eq. 3, providing the rate of propane conversion in terms of propane which was plotted in Fig. 5d. Three CV cycles from 0 to 1.4 V at 50 mV s^{-1} were performed after data collection and the charge passed during the Pt oxide reduction peak on the second cycle was used to normalize for the surface area of the electrode.

VI. Oxidation of CO

1. Constant-potential CO oxidation

To calculate the *CO oxidation rate below 0.5 V, the constant-potential oxidation of solution phase CO was measured as a function of the electrode potential using the procedure shown in Supplementary Fig. 7a. Each experiment was preceded by an electrode cleaning step at 1.4 V, followed by 0.05 V for 20 sec each, repeated 3 times (Supplementary Fig. 1). After cleaning, the MS signal was allowed to stabilize for 600 s at 0.05 V. The potential was then increased to E_{ox} for 360 s. Next, the potential was decreased to 0.05 V to allow the MS signal to stabilize for 600 s. The m/z 16 ionic current during E_{ox} and the following 0.3 V stabilization period were integrated. CO_2 yields were calculated from these integrals, the cumulative CO_2 yields during E_{ox} were plotted in Supplementary Fig. 7b. The CO oxidation rate was calculated for each potential by dividing the CO_2 yield by the 360 s oxidation time and data are shown in Fig. 6c. CO oxidation rates were put in terms of propane oxidation rates by dividing by the stoichiometric coefficient of 3, according to Supplementary Eq. 3, before plotting in Fig. 7a.

2. Oxidation of pre-adsorbed CO

To calculate the *CO oxidation rate at 0.3 V and above, the oxidation of a partial monolayer of CO was measured as a function of the electrode potential using the procedure shown in Fig. 6a. Each experiment was preceded by an electrode cleaning step at 1.4 V, followed by 0.05 V for 20 s each, repeated 3 times (Supplementary Fig. 1). Next, a partial monolayer of CO was adsorbed to the electrode surface by applying 0.3 V to the working electrode for 120 s in a CO-saturated electrolyte. The gas supply was then switched to He to remove CO from the electrolyte while maintaining the potential at 0.3 V for 12 min (first 11 min at 10 sccm He and final 1 min at 1 sccm). Removal of CO from the electrolyte allowed the following oxidation step to involve only

surface bound CO. After removal of CO from the electrolyte, a more oxidative potential E_{ox} was applied for the duration t_{ox}. The potential was then set to 0.3 V to halt CO oxidation and up to 10 min was allowed for the CO_2 signals to decay to the baseline. The quantity of CO_2 produced was plotted vs t_{ox} and E_{ox} as shown in Fig. 6b, and the oxidation rate was determined from a linear fit between 0.1 and 1.0 s. Linear fits of this data are shown in Supplementary Fig. 8 and listed in Supplementary Table 4. The corresponding CO oxidation rates were expressed in terms of propane oxidation rates by divided by the stoichiometric coefficient of 3, according to Supplementary Eq. 3, before plotting in Fig. 7a.

VII. Enhancement of Propane Oxidation Rates via Pulsed Potential Sequences

The propane total oxidation reaction was carried out using the pulsed potential sequence shown in Fig. 8a. Each experiment was preceded by an electrode cleaning step at 1.4 V, followed by 0.05 V for 20 s each, repeated 3 times (Supplementary Fig. 1). After cleaning, the MS signal was allowed to stabilize for 120 s at 0.05 V. After cycling for 360 s (180 cycles), the potential was reduced to 0.3 V which arrested oxidation and allowed the m/z 16 signal to decay to the baseline. The m/z 16 ionic current during potential cycling and the following 0.3 V stabilization period was integrated in 60 s segments to trace CO_2 production over time. CO_2 yields were calculated from these integrals. The cumulative CO_2 yields were divided by the stoichiometric coefficient of 3, according to Supplementary Eq. 3, providing the quantities of consumed propane and were plotted in Fig. 8b. The rate of continuous CO_2 production was determined by fitting linear curves of the CO_2 yield data. This fit is shown in Supplementary Table 5 where the slope is the CO_2 production rate. The rate of propane turnover is calculated by dividing the CO_2 production rate by the stoichiometric coefficient of 3, according to Supplementary Eq. 3. The pulse-potential oxidation of propane was compared to constant-potential oxidation of propane at 0.7 V as shown in Fig. 3b. The propane consumption from pulse-potential oxidation experiments and the constant-potential comparison (from Fig. 3b) were plotted in Fig. 8b.

Comments on the Interpretation of Experimental Results

Between 0.2 and 0.7 V, the rate of conversion was identified as rate limiting. Since adsorption is faster than conversion within this range, this leads to the accumulation of multi-carbon intermediates on the electrode surface. Accumulation of adsorbates in turn decreases the number of free Pt sites available to perform further propane adsorption. The result is a decreased rate of adsorption as the adsorption time is increased (Supplementary Fig. 4). This behavior agrees with the findings of Gilman, Cairns, and coworkers who found that alkane adsorption on Pt in acidic electrolytes was a function of free site population where accumulation of adsorbates reduce the free site population.[8–10] From this information, we can also rationalize why the maximum rate of turnover is observed at 0.7 V. 0.7 V offers the fastest rate of conversion and oxidation without promoting the formation of platinum oxides which inhibit alkane adsorption.

Total Oxidation of Propane Chemical Equation

The electrochemical total oxidation of propane to CO_2 and H^+ is given by,

$$C_3H_8 + 6\ H_2O \rightarrow 3\ CO_2 + 20\ H^+ + 20\ e^-. \qquad \text{(S3)}$$

Supporting Data and Data Fitting

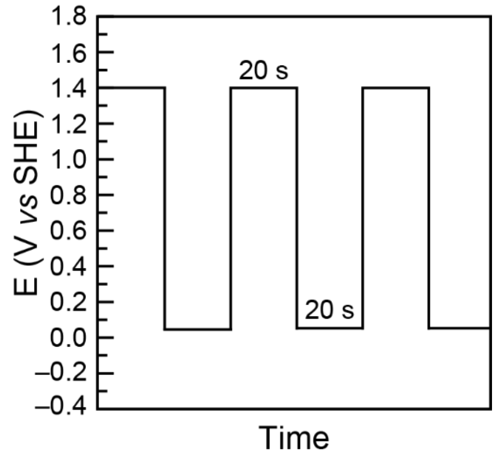

Supplementary Fig. 1. Pulse electrode potential sequence used to clean electrodes before each experiment.

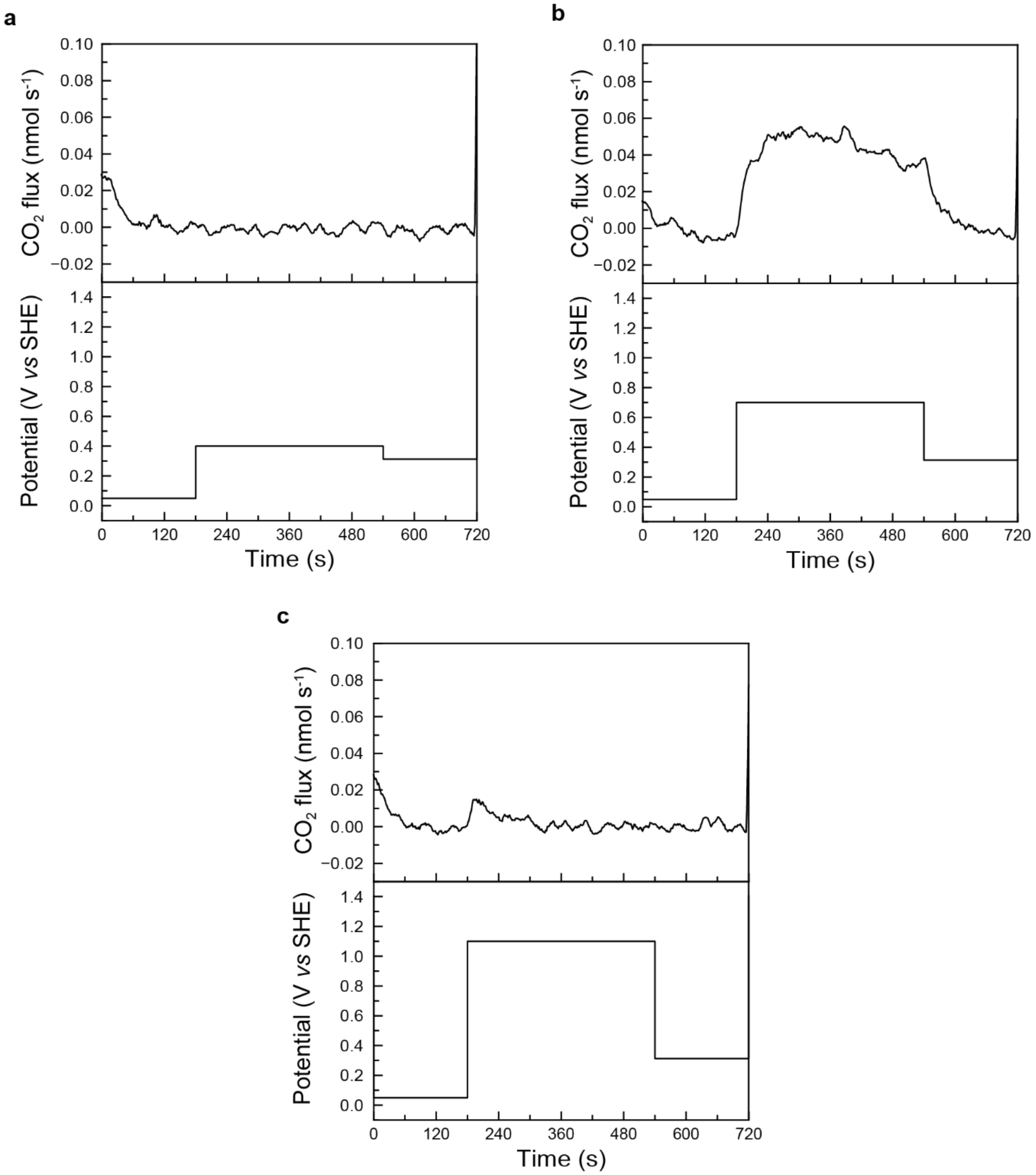

Supplementary Fig. 2. CO_2 flux detected via EC-MS during constant-potential propane oxidation experiments. Representative experiments from $E_{turnover}$ = 0.4, 0.7, and 1.1 V are shown. **(a)** $E_{turnover}$ = 0.4 V. **(b)** $E_{turnover}$ = 0.7 V. **(c)** $E_{turnover}$ = 1.1 V. CO_2 flux was smoothed in Origin 2022b using a 11.2 s window using the Adjacent-Averaging method. Cell resistance = 100.8±0.4 Ω.

Supplementary Table 1. Linear fitting slopes for the turnover data presented in Fig. 3b. No increase in m/z 16 signal was observed at 0.4 V and the negative slope is due to integration and fitting of the slightly decaying baseline signals under conditions of no turnover. This value can be considered zero within the margin of error.

Turnover Potential (V)	Slope (nmol $C^{-1}_{o\text{-}r}$ s^{-1})	R^2
0.4	-0.1345	0.964
0.5	2.1252	0.989
0.6	4.1173	0.993
0.7	5.2295	0.999
0.8	4.0328	0.999
0.9	0.1802	0.912
1.0	0.0931	0.694
1.1	0.0745	0.408

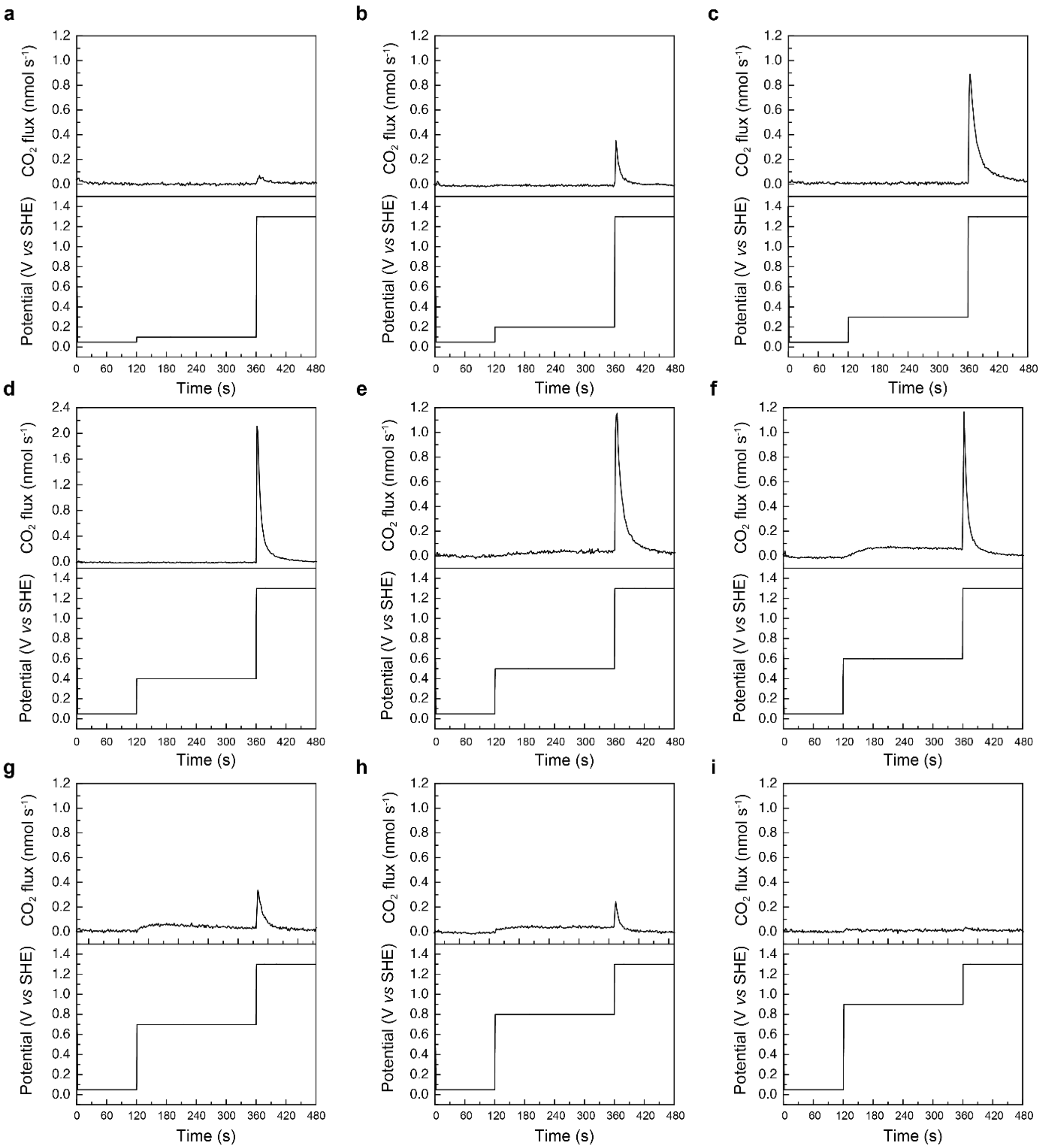

Supplementary Fig. 3. CO_2 flux detected via EC-MS during propane adsorption experiments shown in Supplementary Table 8. Representative experiments from t_{ads} = 240 s are shown. **(a)** E_{ads} = 0.1 V. (Cell resistance = 12.8±2.2 Ω) **(b)** E_{ads} = 0.2 V. (Cell resistance = 17.0±1.6 Ω) **(c)** E_{ads} = 0.3 V. (Cell resistance = 12.8±2.2 Ω) **(d)** E_{ads} = 0.4 V. (Cell resistance = 17.0±1.6 Ω) **(e)** E_{ads} = 0.5 V. (Cell resistance = 12.8±2.2 Ω) **(f)** E_{ads} = 0.6 V. (Cell resistance = 17.0±1.6 Ω) **(g)** E_{ads} = 0.7 V. (Cell resistance = 12.8±2.2 Ω) **(h)** E_{ads} = 0.8 V. (Cell resistance = 17.0±1.6 Ω) **(i)** E_{ads} = 0.9 V. (Cell resistance = 12.8±2.2 Ω).

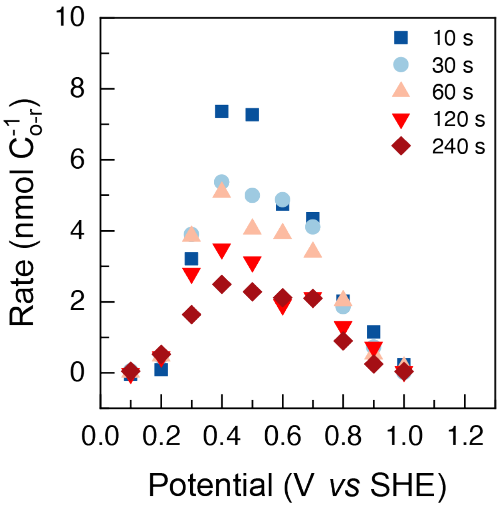

Supplementary Fig. 4. Calculated propane adsorption rates for different adsorption durations. Rate values were obtained by dividing CO_2 yields by the stoichiometric coefficient of 3 for the reaction of propane to CO_2, according to Supplementary Eq. 3.

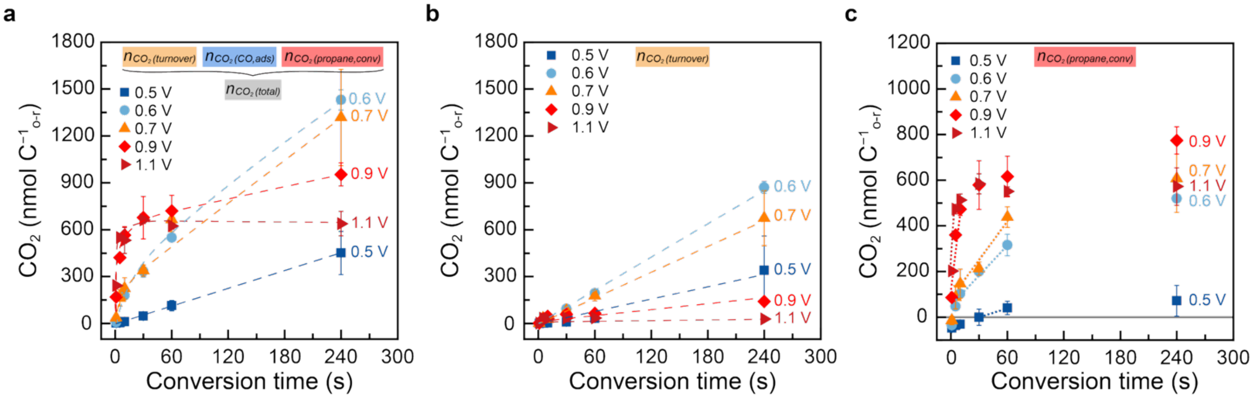

Supplementary Fig. 5. CO_2 yields from conversion experiments above 0.5 V. **(a)** Total CO_2 yields obtained from conversion experiments. Dashed lines are added to guide the eye. **(b)** CO_2 yields from propane turnover used to correct the data presented in Supplementary Fig. 5a. Dashed lines added as guides to the eye. **(c)** Linear fit of CO_2 yield data attributed to the conversion and oxidation of multi-carbon adsorbates. CO_2 yields attributed to propane turnover and pre-adsorbed CO (39.9 nmol $C^{-1}_{o\text{-}r}$) have been subtracted. The CO_2 yields below zero are attributed primarily to the oxidation of this pre-adsorbed CO and thus the negative CO_2 yield values were not considered when performing data fitting as we intend to obtain the rate of only CO_2 production due to the conversion of multi-carbon intermediates. For all plots, error bars represent one standard deviation obtained from data points from at least two separate experiments. Cell resistance = 14.0±0.9 Ω.

Supplementary Table 2. Linear fitting slopes for the fits presented in Supplementary Fig. 5c. Dividing the slope by the stoichiometric coefficient of 3, according to Supplementary Eq. 3, yields the rate of propane conversion as plotted in Fig. 5d.

Oxidation Potential (V)	Slope (nmol $C^{-1}_{o\text{-}r}$ s^{-1})	R^2
0.5	1.38	1
0.6	4.68	0.980
0.7	6.07	0.976
0.9	42.22	0.913
1.1	67.68	1

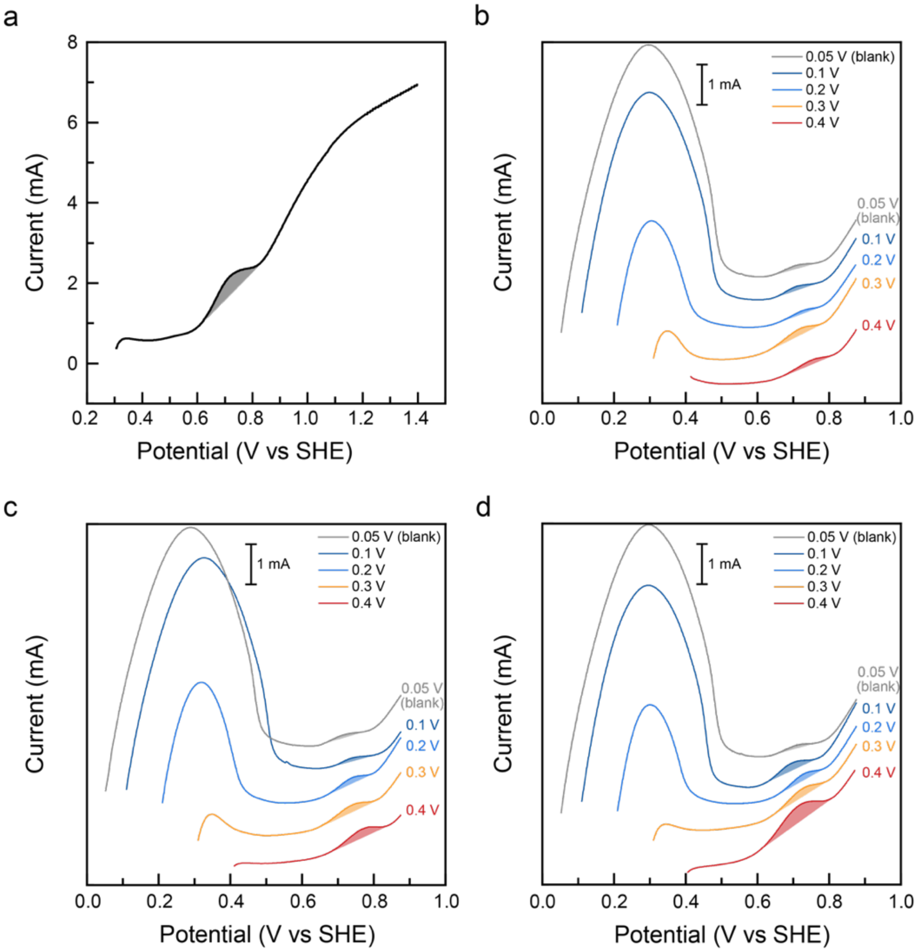

Supplementary Fig. 6. Oxidative linear sweep voltammetric (LSV) stripping for the quantification of *CO formation. The integral of the peak corresponding to *CO oxidation is shown as a shaded area. Scan rate = 100 mV s^{-1}. Each LSV is a single measurement. Data was not iR corrected. **(a)** LSV after propane adsorbed at 0.3 V for 120 s to obtain the *CO correction for multi-carbon adsorbate conversion experiemnts above 0.5 V. Note that experiments using this technique halted the LSV at 0.875 V to only consider CO_2 produced by the oxidation of *CO species corresponding to this peak. The LSV here is carried out until 1.4 V to more clearly show the peak of interest. **(b)** LSVs after t_{conv} = 10 s. **(c)** LSVs after t_{conv} = 30 s. **(d)** LSVs after t_{conv} = 60 s. Cell resistance = 13.7±0.2 Ω.

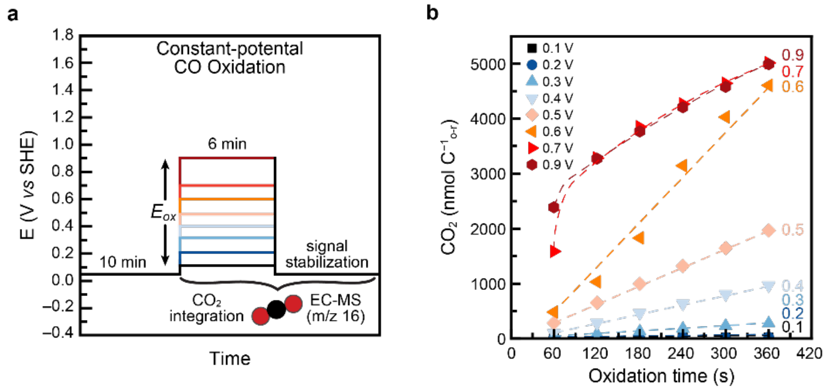

Supplementary Fig. 7. **(a)** Electrode potential program used to study the constant-potential oxidation of solution phase CO. E_{ox} = 0.1, 0.2, 0.3, 0.4, 0.5 V, 0.6, 0.7, 0.9 V. Up to 14 min was allowed for signal stabilization after E_{ox}. **(b)** Cumulative CO_2 yields resulting from the constant-potential oxidation of solution phase CO. Mass transport limitations reached for 0.6 V and above. Each data point was from a single measurement. Dashed lines were added as guides to the eye. Cell resistance = 13.6±0.1 Ω

Supplementary Table 3. Rates of constant-potential solution-phase CO oxidation calculated from the data in Supplementary Fig. 7b.

$E_{turnover}$ (V vs SHE)	0.1	0.2	0.3	0.4	0.5
Rate (nmol s^{-1} $C^{-1}{}_{o-r}$)	0.07	0.09	0.84	3.10	6.25

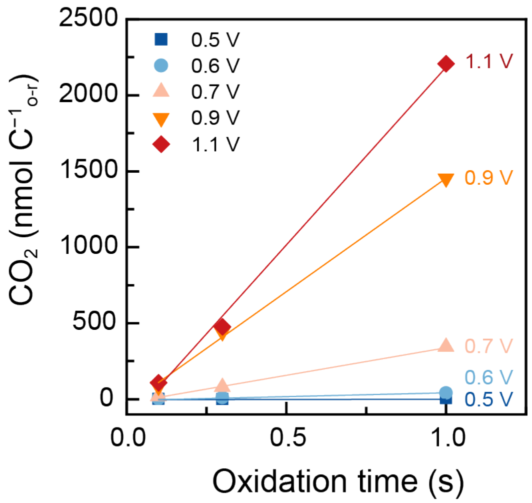

Supplementary Fig. 8. Linear fit of the CO_2 generated during CO oxidation experiments to determine the rate of CO oxidation at different potentials. Each data point is from a single measurement. Cell resistance = 14.5±0.7 Ω

Supplementary Table 4. Linear fitting slopes for the *CO oxidation data presented in Supplementary Fig. 8.

Oxidation Potential (V)	Slope (nmol $C^{-1}_{o\text{-}r}$ s^{-1})	R^2
0.5	4.68	0.774
0.6	42.49	0.999
0.7	366.47	0.999
0.9	1500.98	0.998
1.1	2369.91	0.998

Supplementary Table 5. Linear fitting slopes for the pulse oxidation data presented in Fig. 8b. The slope represents the rate of propane oxidation.

Potential (V)	Slope (nmol $C^{-1}_{o\text{-}r}$ s^{-1})	R^2
0.7	5.230	0.999
E_{ads} = 0.4 (1 s)<br>E_{ox} = 0.9 (1 s)<br>(pulse)	8.657	0.996

Additional Data

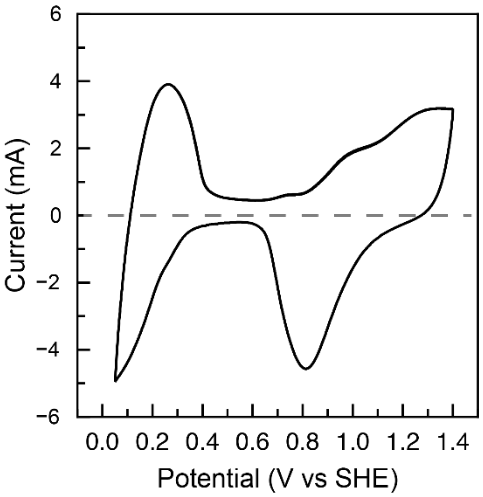

Supplementary Fig. 9. Representative cyclic voltammogram of platinized platinum electrode in He saturated 1 M $HClO_4$ at 60 °C from 0 – 1.4 V vs SHE performed in the EC-MS setup. Scan rate was 50 mV s^{-1}. Cell resistance = 13.7±0.2 Ω. Data was not iR corrected.

Supplementary Table 6. Data set for constant-potential propane oxidation (turnover) used to plot Fig. 3b. Cumulative CO_2 produced calculate from the MS m/z 16 data integrated from time zero to $t_{turnover}$. No increase in m/z 16 signal was observed at 0.4 V. Cell resistance = 100.8±0.4 Ω.

**CO_2 yield (nmol $C^{-1}_{o\text{-}r}$)**								
$t_{turnover}$ (s)	$E_{turnover}$ (V vs SHE)							
	0.4	0.5	0.6	0.7	0.8	0.9	1.0	1.1
60	-	89.7	181.7	198.4	160.3	80.3	68.5	64.2
120	-	153.2	494.5	529.0	412.5	105.9	82.1	96.2
180	-	305.8	773.4	861.3	668.2	107.9	94.4	96.0
240	-	450.2	1012.0	1177.6	902.8	125.6	102.2	97.9
300	-	548.3	1209.1	1450.0	1109.4	134.6	95.7	94.7
360	-	716.4	1434.5	1779.0	1389.0	135.2	97.9	95.9

Supplementary Table 7. CO_2 yields for propane adsorption experiments. **Bold** values indicate averaged values; *italicized* values indicate standard deviation values. This data set was run in E_{ads} sub-sets in the order: 0.5[a], 0.7, 0.1, 0.6, 0.9, 0.3, 0.4, 0.2, 0.8, 0.5[b] V. Within each sub-set, experiments t_{ads} followed the order: 0, 30, 5, 1, 10, 60, 0 s. Each data sub-set was book-ended by an experiments of E_{ads} = 0.3 V t_{ads} = 60 s and may be used to evaluate electrode stability. The average value listed in Adsorption Data Set 1 Blanks for a specific E_{ads} data sub-set was subtracted from its respective E_{ads} data sub-set to yield the values listed in Adsorption Data Set 1. Data plotted in Supplementary Fig. 10a. Adsorption at short time points was minimal and within error of blank runs. Negative values may thus be produced upon blank subtraction. These values can be considered zero within the margin of error. Blank cells indicate that no data was collected for the specified parameters. Cell resistance = 100.3±0.4 Ω.

Adsorption Data Set 1 – CO_2 yield (nmol $C^{-1}_{o\text{-}r}$) [blank subtracted]

t_{ads} (s)	E_{ads} (V vs SHE)								
	0.1	0.2	0.3	0.4	0.5	0.6	0.7	0.8	0.9
1	13.2	2.3	-12.3		**1.2**	-0.7	3.6	-8.9	9.4
5	0.7	15.7	-12.1	37.8	**75.2**	19.5	80.3	48.4	29.8
10	8.0	4.6	35.0	185.0	**230.7**	209.2	178.2	66.4	49.2
30	12.6	17.3	422.6	724.6	**607.3**	584.9	436.6	173.0	76.4
60	8.3	80.4	**863.1**	1190.5	**979.7**	894.1	768.0	303.1	127.0

Adsorption Data Set 1 Blanks – CO_2 yield (nmol $C^{-1}_{o\text{-}r}$) [t_{ads} = 0 s]

t_{ads} (s)	E_{ads} (V vs SHE)									
	0.1	0.2	0.3	0.4	0.5[a]	0.6	0.7	0.8	0.9	0.5[b]
0	55.2	43.9	41.8	33.6	57.8	43.4	44.3	28.8	49.4	31.6
0	52.8	31.6	54.5	32.8	48.9	36.6	50.3	31.4	47.4	30.2
average	**54.0**	**37.8**	**48.1**	**33.2**	**53.4**	**40.0**	**47.3**	**30.1**	**48.4**	**30.9**
Std. Dev.	*1.7*	*8.7*	*8.9*	*0.6*	*6.3*	*4.8*	*4.2*	*1.8*	*1.4*	*1.0*

Adsorption Data Set 1 Replicates – CO_2 yield (nmol $C^{-1}_{o\text{-}r}$) [blank subtracted]

t_{ads} (s)	E_{ads} (V vs SHE)			
	0.5[a]	0.5[b]	average	Std. Dev
1	-33.1	35.5	**1.2**	*48.5*
5	38.5	111.8	**75.2**	*51.8*
10	149.9	311.5	**230.7**	*114.3*
30	409.0	805.6	**607.3**	*280.4*
60	699.2	1260.1	**979.7**	*396.6*

Adsorption Data Set 1 Replicates E_{ads} = 0.3 V t_{ads}= 60 s replicates – CO_2 yield (nmol $C^{-1}_{o\text{-}r}$) [blank subtracted]

t_{ads} (s)	E_{ads} = 0.3 V t_{ads}= 60 s replicate for corresponding E_{ads} (V vs SHE) data set									
	0.1	0.2	0.3	0.4	0.5[a]	0.6	0.7	0.8	0.9	0.5[b]
60	1001.6	782.5	893.8	833.1	527.9	1028.0	994.5		930.1	622.9
60	1024.9	871.5	832.7	951.8	625.7		1013.0	571.9	953.5	1077.1
Average	**863.1**									
Std. dev.	*171.3*									

Supplementary Table 8. CO_2 yields for propane adsorption experiments. Data Set 2 is comprised of two data sub-sets. The data sub-set 1 is indicated using the dark grey shading (Cell resistance = 17.0±1.6 Ω). Data sub-set 2 is indicated using the light grey shading (Cell resistance = 12.8±2.2 Ω). The average values in Data Set 2 blanks was subtracted from its respective sub-set data to yield the data listed in Data Set 2. Unshaded cells are averages using both data sub-sets. **Bold** values indicate averaged values; *italicized* values indicate standard deviation values. Data plotted in Supplementary Fig. 10b. Adsorption at short time points, the most oxidative potentials, and the most reductive potentials was minimal and within error of blank runs. Negative values may thus be produced upon blank subtraction. These values can be considered zero within the margin of error. Blank cells indicate that no data was collected for the specified parameters.

Adsorption Data Set 2 – CO_2 yield (nmol $C^{-1}_{o\text{-}r}$) [blank subtracted]

t_{ads} (s)	E_{ads} (V vs SHE) 0.1	0.2	0.3	0.4	0.5	0.6	0.7	0.8	0.9	1.0
10	-39.6	18.2	153.3	**249.5**	278.1	134.6	77.6	31.6	-35.8	-15.3
30	-40.5	43.5	456.5	**459.1**	414.8	448.8	524.9	169.8	-23.7	-23.7
60	-13.9	78.7	**830.7**	**861.1**	924.5	800.3	671.2	302.9	28.8	5.6
120	-34.1	143.4	1242.9	**1332.4**	1163.0	939.4	1156.3	560.8	35.9	-11.9
240	13.9	284.7	1493.4	**1965.8**	2346.4	**2011.4**	1382.2	931.4	85.1	-34.8

Adsorption Data Set 2 Replicates – CO_2 yield (nmol $C^{-1}_{o\text{-}r}$) [blank subtracted]

t_{ads} (s)	E_{ads} (V vs SHE) 0.4	0.4	Average 0.4 V	Std. Dev. 0.4 V	0.6	0.6	Average 0.6 V	Std. Dev. 0.6 V
10	257.4	241.7	**249.5**	*11.1*				
30	492.6	425.5	**459.1**	*47.4*				
60	825.1	897.2	**861.1**	*51.0*				
120	1274.9	1389.8	**1332.4**	*81.2*				
240	2182.4	1749.1	**1965.8**	*306.4*	2137.9	1884.9	**2011.4**	*178.9*

Adsorption Data Set 2 Replicates E_{ads} = 0.3 V t_{ads}= 60 s – CO_2 yield (nmol $C^{-1}_{o\text{-}r}$) [blank subtracted]

t_{ads} (s)	E_{ads} (V vs SHE) 0.3	0.3	0.3	0.3	0.3	0.3	0.3	0.3	0.3	Average 0.3 V	Std. Dev. 0.3 V
10											
30											
60	834.4	740.0	700.9	907.4	993.3	761.9	815.8	924.8	797.6	**830.7**	*95.1*
120											
240											

Adsorption Data Set 2 Blanks – CO_2 yield (nmol $C^{-1}_{o\text{-}r}$) [t_{ads} = 0 s]

t_{ads} (s)	E_{ads} (V vs SHE) A	B	C	D	E	F	G	H	Average	Std. Dev.
0	10.3	10.7	11.3	10.7					10.7	0.4
0	68.4	33.0	8.7	56.7					41.7	22.9

Supplementary Table 9. CO_2 yields for propane adsorption experiments. Data Set 3 is comprised of three data sub-sets. The data sub-sets 1, 2, and 3 are indicated with light, medium, and dark grey shading, respectively. The average values in Data Set 3 Blanks were subtracted from the respective sub-set data to yield the data listed in Data Set 3. Unshaded cells are averages using multiple data sub-sets. **Bold** values indicate averaged values; *italicized* values indicate standard deviation values. Data plotted in Supplementary Fig. 10c. Adsorption at short time points, the most oxidative potentials, and the most reductive potentials was minimal and within error of blank runs. Negative values may thus be produced upon blank subtraction. These values can be considered zero within the margin of error. Blank cells indicate that no data was collected for the specified parameters. Cell resistance for data sub-sets 1,2, and 3 were 12.20±0.03 Ω, 12.01±0.03 Ω, or 12.16±0.11 Ω, respectively.

Adsorption Data Set 3 – CO_2 yield (nmol $C^{-1}_{o\text{-}r}$) [blank subtracted]

t_{ads} (s)	E_{ads} (V vs SHE)										
	0.1	0.2	0.25	0.3	0.4	0.5	0.6	0.7	0.8	0.9	1.0
10	-2.6	-7.7	69.4	165.4	179.1	61.8	100.6	80.9	58.0	-1.0	-1.2
30	-4.9	45.2	**134.3**	255.2	**389.2**	**336.7**	**171.7**	154.5	110.1	-5.2	-17.6
60	-14.5	94.7	274.4	**464.5**	480.9	403.7	453.5	483.8	96.3	24.6	
120	-7.1	185.7	725.7	1058.1	1059.3	**490.6**	335.8	**374.9**	458.6	8.2	9.7
240	6.5	443.6	840.7	1362.8	888.1	**686.5**	1597.1	359.8	235.9	50.4	-0.7
360	25.8	791.5	1385.5	**1751.2**	**1201.9**	1139.9	1043.8	274.3		**23.0**	-14.8

Adsorption Data Set 3 Replicates – CO_2 yield (nmol $C^{-1}_{o\text{-}r}$) [blank subtracted]

Replicate	t_{ads} (s) @ E_{ads} (V vs SHE) condition										
	30 @ 0.25	60 @ 0.3	360 @ 0.3	30 @ 0.4	360 @ 0.4	30 @ 0.5	120 @ 0.5	240 @ 0.5	30 @ 0.6	120 @ 0.7	360 @ 0.9
A	148.5	625.1	1966.7	451.1	1154.4	404.3	543.1	854.6	91.5	334.5	42.6
B	120.1	696.6	1535.7	327.3	1249.3	269.1	438.2	518.5	252.0	415.3	3.4
C		439.5									
D		306.0									
E		448.9									
F		599.3									
G		493.5									
H		412.1									
I		159.4									
Average	**134.3**	**464.5**	**1751.2**	**389.2**	**1201.9**	**336.7**	**490.6**	**686.5**	**171.7**	**374.9**	**23.0**
Std. Dev.	20.0	166.0	304.7	87.5	67.1	95.6	74.2	237.6	113.5	57.1	27.7

Adsorption Data Set 3 Blanks – CO_2 yield (nmol $C^{-1}_{o\text{-}r}$) [t_{ads} = 0 s]

t_{ads} (s)	E_{ads} (V vs SHE)							
	A	B	C	D	E	F	Average	Std. Dev.
0	6.2	8.7	19.0				11.3	6.8
0	36.8						36.8	
0	26.2	17.9	3.9	31.4	18.3	23.7	20.3	9.5

Supplementary Table 10. Averaged CO_2 yield data from Supplementary Tables 7, 8, and 9 Adsorption Data Sets 1, 2, and 3. This data was plotted and presented as Fig. 4b. Adsorption at short time points, the most oxidative potentials, and the most reductive potentials was minimal and within error of blank runs. Negative values may thus be produced upon blank subtraction and the averaging of multiple data sets. These values can be considered zero within the margin of error. Blank cells indicate that no data was collected for the specified parameters.

Adsorption Data Set 1, 2, and 3 Average – CO_2 yield (nmol $C^{-1}_{o\text{-}r}$) [blank subtracted]

t_{ads} (s)	E_{ads} (V vs SHE)									
	0.1	0.2	0.3	0.4	0.5	0.6	0.7	0.8	0.9	1.0
1	13.2	2.3	-12.3		1.2	-0.7	3.6	-8.9	9.4	
5	0.7	15.7	-12.1	37.8	75.2	19.5	80.3	48.4	29.8	
10	-11.4	5.0	117.9	204.5	190.2	148.1	112.2	52.0	4.2	-8.3
30	-10.9	35.4	378.1	524.3	452.9	401.8	372.0	151.0	15.8	-20.6
60	-6.7	84.6	734.9	844.2	769.3	716.0	641.0	234.1	60.1	5.6
120	-20.6	164.5	1150.5	1195.9	826.8	637.6	765.6	509.7	22.0	-1.1
240	10.2	364.1	1428.1	1426.9	1516.4	1804.3	871.0	583.6	67.8	-17.7
360	25.8	791.5	1751.2	1201.9	1139.9	1043.8	274.3		23.0	-14.8

Adsorption Data Set 1, 2, and 3 Standard Deviation – CO_2 yield (nmol $C^{-1}_{o\text{-}r}$) [blank subtracted]

t_{ads} (s)	E_{ads} (V vs SHE)									
	0.1	0.2	0.3	0.4	0.5	0.6	0.7	0.8	0.9	1.0
1										
5										
10	25.0	12.9	72.0	39.1	113.7	55.6	57.2	18.2	42.7	10.0
30	27.0	15.7	107.8	176.9	139.3	210.5	193.4	35.4	53.3	4.3
60	13.0	8.8	237.1	355.1	317.8	232.1	144.5	119.4	58.0	
120	19.1	29.9	130.7	193.1	475.5	426.8	552.5	72.3	19.6	15.3
240	5.2	112.4	92.4	762.1	1173.7	292.9	722.9	491.8	24.5	24.2
360										

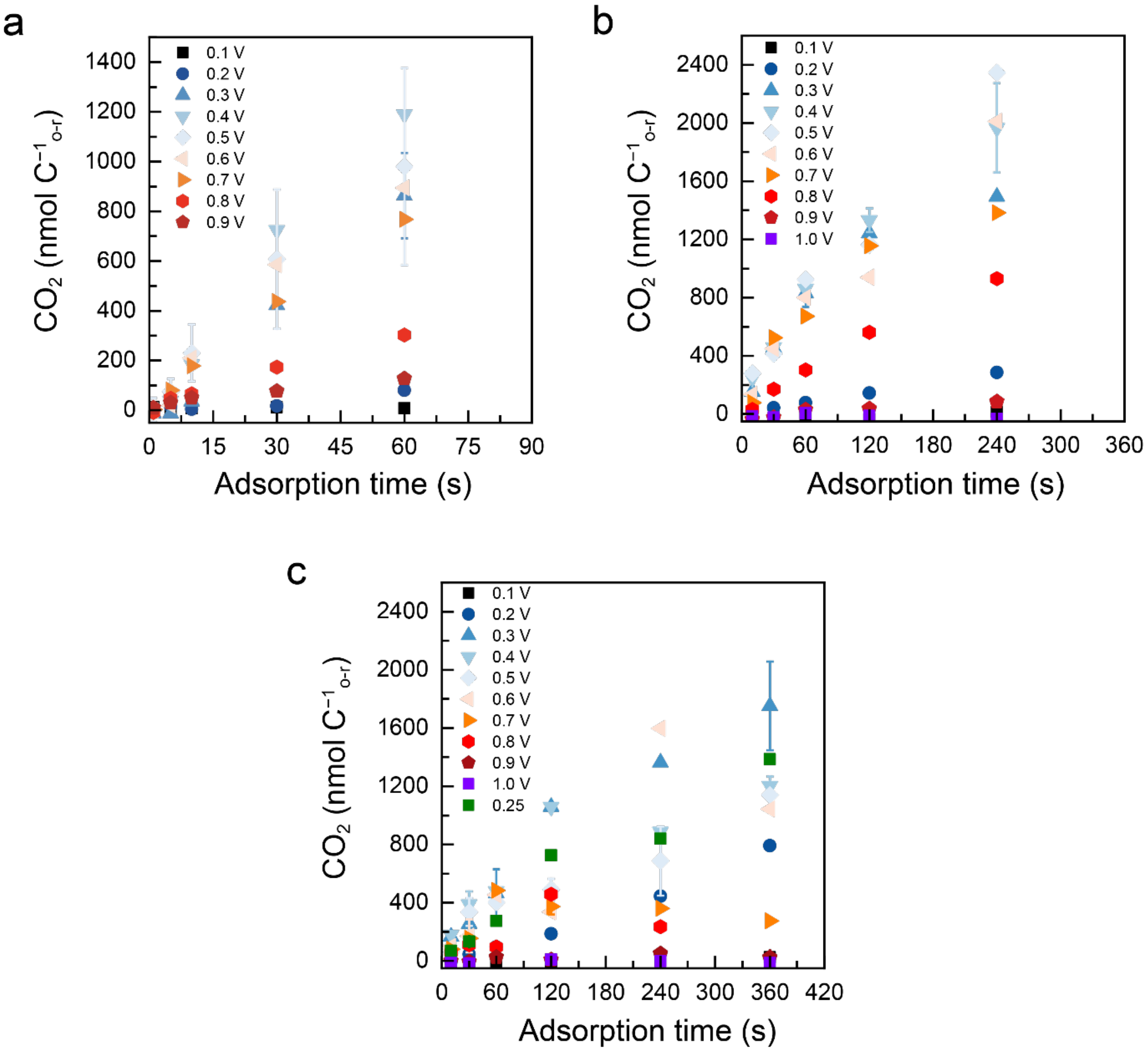

Supplementary Fig. 10. CO_2 yield data from Adsorption experiments. (a) CO_2 yield data from Supplementary Table 7 Adsorption Data Set 1 with error bars from Adsorption Data Set 1 Replicates. Cell resistance = 100.3±0.4 Ω. (b) CO_2 yield data from Supplementary Table 8 Adsorption Data Set 2 with error bars from Adsorption Data Set 2 Replicates. Cell resistance = 17.0±1.6 Ω and 12.8±2.2 Ω (c) CO_2 yield data from Supplementary Table 9 Adsorption Data Set 3 with error bars from Adsorption Data Set 3 Replicates. Cell resistance = 12.20±0.03 Ω, 12.01±0.03 Ω, and 12.16±0.11 Ω.

Supplementary Table 11. Data Set 1 for propane conversion experiments. Data that was considered an outlier compared to replicate measurements and not considered in the averaged data set are struck-through. Blank cells indicate that no data was collected for the specified parameters. Cell resistance = 13.2±0.3 Ω.

**Conversion Data Set 1 – Total CO_2 yield (nmol $C^{-1}_{o\text{-}r}$)**					
t_{conv} (s)	E_{conv} (V vs SHE)				
	0.5	0.6	0.7	0.9	1.1
1		8.1		~~3.6~~	
5					
10		189.7		603.7	
30		360.1		824.3	
60		550.4		837.9	
240		1476.6		1054.9	

**Conversion Data Set 1 – Constant-potential Turnover CO_2 yield (nmol $C^{-1}_{o\text{-}r}$)**					
t_{conv} (s)	E_{conv} (V vs SHE)				
	0.5	0.6	0.7	0.9	1.1
1		1.82		~~1.5~~	
5					
10		35.5		57.2	
30		94.9		79.8	
60		213.4		94.1	
240		897.6		154.9	

**Conversion Data Set 1 – Turnover subtracted total CO_2 yield (nmol $C^{-1}_{o\text{-}r}$)**					
t_{conv} (s)	E_{conv} (V vs SHE)				
	0.5	0.6	0.7	0.9	1.1
1		6.3		~~2.1~~	
5					
10		154.2		546.5	
30		265.2		744.5	
60		337.0		743.7	
240		579.0		900.0	

**Conversion Data Set 1 – CO_2 from *CO (nmol $C^{-1}_{o\text{-}r}$)**
A
54.0

Supplementary Table 12. Data Set 2 for propane conversion experiments. Data that was considered an outlier compared to replicate measurements and not considered in the averaged data set are struck-through. Blank cells indicate that no data was collected for the specified parameters. Cell resistance = 13.4±0.1 Ω.

**Conversion Data Set 2 – Total CO_2 yield (nmol $C^{-1}_{o\text{-}r}$)**					
t_{conv} (s)	E_{conv} (V vs SHE)				
	0.5	0.6	0.7	0.9	1.1
1				179.5	231.6
5				434.3	558.0
10				527.2	557.7
30				560.2	676.6
60				683.9	608.9
240				904.6	694.1

**Conversion Data Set 2 – Constant-potential Turnover CO_2 yield (nmol $C^{-1}_{o\text{-}r}$)**					
t_{conv} (s)	E_{conv} (V vs SHE)				
	0.5	0.6	0.7	0.9	1.1
1				3.3	1.2
5				25.5	31.3
10				34.6	25.6
30				44.4	23.0
60				58.6	33.4
240				140.3	25.5

**Conversion Data Set 2 – Turnover subtracted total CO_2 yield (nmol $C^{-1}_{o\text{-}r}$)**					
t_{conv} (s)	E_{conv} (V vs SHE)				
	0.5	0.6	0.7	0.9	1.1
1				176.2	230.4
5				408.8	526.7
10				492.6	532.1
30				515.8	653.6
60				625.3	575.5
240				764.2	668.6

**Conversion Data Set 2 – CO_2 from *CO (nmol $C^{-1}_{o\text{-}r}$)**
A
38.5

Supplementary Table 13. Data Set 3 for propane conversion experiments. Data that was considered an outlier compared to replicate measurements and not considered in the averaged data set are struck-through. Blank cells indicate that no data was collected for the specified parameters. Cell resistance = 13.2±0.1 Ω.

Conversion Data Set 3 – Total CO_2 yield (nmol $C^{-1}_{o\text{-}r}$)

t_{conv} (s)	E_{conv} (V vs SHE)				
	0.5	0.6	0.7	0.9	1.1
1				158.7	250.8
5			199.3		
10					~~438.3~~
30			363.3		
60				622.9	637.1
240			~~747.2~~	894.1	582.8

Conversion Data Set 3 – Constant-potential Turnover CO_2 yield (nmol $C^{-1}_{o\text{-}r}$)

t_{conv} (s)	E_{conv} (V vs SHE)				
	0.5	0.6	0.7	0.9	1.1
1				2.8	1.5
5			39.8		
10					~~30.6~~
30			93.0		
60				59.4	36.4
240			~~687.9~~	132.8	32.4

Conversion Data Set 3 – Turnover subtracted total CO_2 yield (nmol $C^{-1}_{o\text{-}r}$)

t_{conv} (s)	E_{conv} (V vs SHE)				
	0.5	0.6	0.7	0.9	1.1
1				156.0	249.3
5			159.4		
10					~~407.7~~
30			270.3		
60				563.5	600.7
240			~~59.3~~	761.2	550.5

Conversion Data Set 3 – CO_2 from *CO (nmol $C^{-1}_{o\text{-}r}$)

A
36.2

Supplementary Table 14. Data Set 4 for propane conversion experiments. Data that was considered an outlier compared to replicate measurements and not considered in the averaged data set are struck-through. Blank cells indicate that no data was collected for the specified parameters. Cell resistance = 14.9±0.6 Ω.

Conversion Data Set 4 – Total CO_2 yield (nmol $C^{-1}_{o\text{-}r}$)

t_{conv} (s)	E_{conv} (V vs SHE)				
	0.5	0.6	0.7	0.9	1.1
1	1.5		50.3		
5					541.4
10			272.2		
30					646.3
60	152.6		718.5		
240					

Conversion Data Set 4 – Constant-potential Turnover CO_2 yield (nmol $C^{-1}_{o\text{-}r}$)

t_{conv} (s)	E_{conv} (V vs SHE)				
	0.5	0.6	0.7	0.9	1.1
1	0.3		2.7		
5					34.9
10			31.8		
30					44.7
60	30.6		199.9		
240					

Conversion Data Set 4 – Turnover subtracted total CO_2 yield (nmol $C^{-1}_{o\text{-}r}$)

t_{conv} (s)	E_{conv} (V vs SHE)				
	0.5	0.6	0.7	0.9	1.1
1	1.2		47.6		
5					506.5
10			240.4		
30					601.6
60	122.0		518.6		
240					

Conversion Data Set 4 – CO_2 from *CO (nmol $C^{-1}_{o\text{-}r}$)

A
48.3

Supplementary Table 15. Data Set 5 for propane conversion experiments. Data that was considered an outlier compared to replicate measurements and not considered in the averaged data set are struck-through. Blank cells indicate that no data was collected for the specified parameters. Cell resistance = 15.5±0.2 Ω.

Conversion Data Set 5 – Total CO_2 yield (nmol $C^{-1}_{o\text{-}r}$)

t_{conv} (s)	E_{conv} (V vs SHE)				
	0.5	0.6	0.7	0.9	1.1
1	0.2		22.5		
5	2.3		125.8		
10	7.1		175.5		
30	28.2		312.9		
60	98.3		609.6	584.7	
240	352.6		1100.9		

Conversion Data Set 5 – Constant-potential Turnover CO_2 yield (nmol $C^{-1}_{o\text{-}r}$)

t_{conv} (s)	E_{conv} (V vs SHE)				
	0.5	0.6	0.7	0.9	1.1
1	0.1		1.0		
5	1.2		25.1		
10	1.3		25.0		
30	5.4		71.5		
60	31.5		154.6	51.4	
240	183.6		549.2		

Conversion Data Set 5 – Turnover subtracted total CO_2 yield (nmol $C^{-1}_{o\text{-}r}$)

t_{conv} (s)	E_{conv} (V vs SHE)				
	0.5	0.6	0.7	0.9	1.1
1	0.1		21.5		
5	1.1		100.7		
10	5.7		150.5		
30	22.8		241.4		
60	66.7		455.0	533.3	
240	169.1		551.6		

Conversion Data Set 5 – CO_2 from *CO (nmol $C^{-1}_{o\text{-}r}$)

A
48.9

Supplementary Table 16. Data Set 6 for propane conversion experiments. Data that was considered an outlier compared to replicate measurements and not considered in the averaged data set are struck-through. Blank cells indicate that no data was collected for the specified parameters. Cell resistance = 14.5±0.2 Ω.

Conversion Data Set 6 – Total CO_2 yield (nmol $C^{-1}_{o\text{-}r}$)

t_{conv} (s)	E_{conv} (V vs SHE)				
	0.5	0.6	0.7	0.9	1.1
1		7.1			
5	3.1	89.4			
10	14.0	168.0			
30	66.0	307.2			
60		547.7		843.4	
240	548.2	1385.1			

Conversion Data Set 6 – Constant-potential Turnover CO_2 yield (nmol $C^{-1}_{o\text{-}r}$)

t_{conv} (s)	E_{conv} (V vs SHE)				
	0.5	0.6	0.7	0.9	1.1
1		2.7			
5	1.1	14.6			
10	4.2	35.7			
30	14.2	91.9			
60		171.3		79.8	
240	495.7	844.2			

Conversion Data Set 6 – Turnover subtracted total CO_2 yield (nmol $C^{-1}_{o\text{-}r}$)

t_{conv} (s)	E_{conv} (V vs SHE)				
	0.5	0.6	0.7	0.9	1.1
1		4.4			
5	2.1	74.8			
10	9.8	132.3			
30	51.8	215.3			
60		376.4		763.6	
240	52.5	541.0			

Conversion Data Set 6 – CO_2 from *CO (nmol $C^{-1}_{o\text{-}r}$)

A
27.3

Supplementary Table 17. Data Set 7 for propane conversion experiments. Data that was considered an outlier compared to replicate measurements and not considered in the averaged data set are struck-through. Blank cells indicate that no data was collected for the specified parameters. Cell resistance = 14.3±0.0 Ω.

Conversion Data Set 7 – Total CO_2 yield (nmol $C^{-1}_{o\text{-}r}$)

t_{conv} (s)	E_{conv} (V vs SHE)				
	0.5	0.6	0.7	0.9	1.1
1					
5				406.1	
10					597.8
30				645.4	
60	92.0			732.1	
240			1536.4	958.7	

Conversion Data Set 7 – Constant-potential Turnover CO_2 yield (nmol $C^{-1}_{o\text{-}r}$)

t_{conv} (s)	E_{conv} (V vs SHE)				
	0.5	0.6	0.7	0.9	1.1
1					
5				29.8	
10					39.8
30				50.3	
60	35.3			57.4	
240			797.9	131.6	

Conversion Data Set 7 – Turnover subtracted total CO_2 yield (nmol $C^{-1}_{o\text{-}r}$)

t_{conv} (s)	E_{conv} (V vs SHE)				
	0.5	0.6	0.7	0.9	1.1
1					
5				376.3	
10					558.1
30				595.1	
60	56.7			674.7	
240			738.5	827.1	

Conversion Data Set 7 – CO_2 from *CO (nmol $C^{-1}_{o\text{-}r}$)

A
26.3

Supplementary Table 18. Averaged turnover subtracted CO_2 yields for propane Conversion Data Set 1-7 and standard deviation for turnover subtracted CO_2 yields for propane Conversion Data Set 1-7 listed in Supplementary Tables 11-17. Data that was considered an outlier, represented by strike-through, are not considered in these average and standard deviation values. **Bold** values indicate averaged values; *italicized* values indicate standard deviation values. Blank cells indicate that no data was collected for the specified parameters.

All Conversion Data – Average CO_2 turnover subtracted CO_2 yield (nmol C^{-1}_{o-r})

t_{conv} (s)	E_{conv} (V vs SHE)				
	0.5	0.6	0.7	0.9	1.1
1	**0.7**	**5.3**	**34.5**	**166.1**	**239.8**
5	**1.6**	74.8	**130.1**	**392.5**	**516.6**
10	**7.8**	**143.2**	**195.5**	**519.5**	**545.1**
30	**37.3**	**240.3**	**255.8**	**618.5**	**627.6**
60	**81.8**	**356.7**	**486.8**	**655.6**	**588.1**
240	**110.8**	**560.0**	**645.1**	**813.1**	**609.5**

All Conversion Data – standard deviation of CO_2 turnover subtracted CO_2 yield (nmol C^{-1}_{o-r})

t_{conv} (s)	E_{conv} (V vs SHE)				
	0.5	0.6	0.7	0.9	1.1
1	*0.8*	*1.3*	*18.5*	*14.3*	*13.3*
5	*0.7*		*41.5*	*23.0*	*14.3*
10	*2.9*	*15.4*	*63.6*	*38.1*	*18.4*
30	*20.5*	*35.3*	*20.4*	*116.1*	*36.8*
60	*35.2*	*27.8*	*45.0*	*86.6*	*17.8*
240	*82.4*	*26.9*	*132.1*	*65.4*	*83.6*

All Conversion Data – Average and standard deviation CO_2 from *CO (nmol C^{-1}_{o-r})

Average	Std. Dev.
**39.9**	*10.9*

Supplementary Table 19. CO_2 yields for propane conversion below 0.5 V calculated from LSV charge integration. Experimental procedure shown in Fig. 5e. **Bold** values indicate averaged values; *italicized* values indicate standard deviations. Data plotted in Supplementary Fig. 11. Cell resistance = 13.7±0.2 Ω.

Conversion Below 0.5 V – CO_2 yield (nmol $C^{-1}_{o\text{-}r}$)					
t_{conv} (s)	E_{conv} (V vs SHE)				
	0.1	0.2	0.3	0.4	Blank
0					**87.3**
10	86.6	90.7	**160.8**	12.5	
30	69.3	146.8	164.8	117.5	
60	119.7	154.1	210.3	243.7	

Conversion Below 0.5 V Replicates – CO_2 yield (nmol $C^{-1}_{o\text{-}r}$)								
t_{conv} (s)	E_{conv} (V vs SHE)							
	0.3	0.3	Average 0.4 V	Std. Dev. 0.4 V	blank	blank	Average blank	Std. Dev. blank
10	162.9	158.7	**160.8**	*2.9*				
0 (Blank)					77.5	97.2	**87.3**	*13.9*

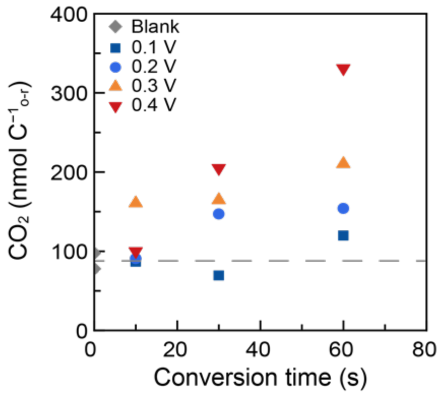

Supplementary Fig. 11. Plotted CO_2 yield data from Supplementary Table 19. Yields determined by integrating the LSV *CO peaks in Supplementary Fig. 6. Experiments followed the potential program shown in Fig. 5e. Grey dashed line indicates the average blank value (87.3 nmol $C^{-1}_{o\text{-}r}$). Each data point was from a single measurement. Cell resistance = 13.7±0.2 Ω.

In the ‘Rate of Multi-carbon Adsorbate Conversion section’, conversion experiments above 0.5 V required correction for the propane converted and oxidized due to continuous turnover. To assess the accuracy of these corrections, we analyzed whether the carbon balance closes. If the correction and analysis is accurate, then the number of carbon atoms released from the oxidation of converted and unconverted propane should sum to the total adsorbed amount, which is on average 336 nmol C^{-1}_{o-r} propane for propane adsorbed for 120 s at 0.3 V. To check this, we performed additional experiments that have been added to the SI (Supplementary Fig. 12). These experiments were variations of the conversion experiments shown in Fig. 5a where the potential was stepped to 1.3 V directly following the completion of a 240 s conversion step at 0.7 V (Supplementary Fig. 12a) or 0.9 V (Supplementary Fig. 12b), to quantify the number of unconverted carbon atoms that remain on the surface. An increase in the m/z 16 EC-MS signal was observed upon the application of 1.3 V indicating the oxidation of unconverted propane. A smaller quantity of unconverted propane was oxidized after conversion at 0.9 V vs 0.7 V.

For each experiment, the propane oxidized during conversion and the unconverted propane oxidized at 1.3 V were summed. Once corrections were made for the propane oxidized due to continuous turnover at the conversion potential, we estimated that 430 and 408 nmol C^{-1}_{o-r} propane were adsorbed during the initial 0.3 V 120 s adsorption step for conversion at 0.7 (Supplementary Fig. 12a) and 0.9 V (Fig. 12b), respectively. These values are within error of the expected 336 nmol C^{-1}_{o-r} for propane adsorption at 0.3 V for 120 s.

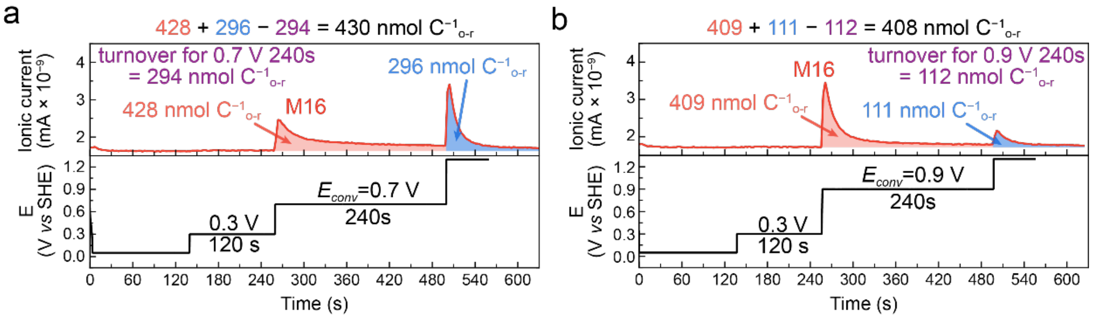

Supplementary Fig. 12. Modified potential program and m/z 16 EC-MS ionic current response for a multi-carbon adsorbate conversion experiment. Following adsorption at 0.3 V for 120 s and conversion at E_{conv} for 240 s, 1.3 V was applied to the electrode to oxidatively remove residual adsorbates. Red shading indicates the integrated area associated with the application of E_{conv}. Blue shading indicates the integrated area associated with the application of 1.3 V. The quantity of propane oxidized during each peak was calculated from the integrated ionic current area and is labeled. The quantity of propane oxidized due to continuous oxidation (turnover) at E_{conv} is noted in purple and subtracted from the total oxidized propane. Both measurements were performed a single time. **(a)** E_{conv} = 0.7 V. Cell resistance = 35.6 Ω. **(b)** E_{conv} = 0.9 V. Cell resistance = 35.6 Ω.

EC-MS Calibration Curves and Data Normalization

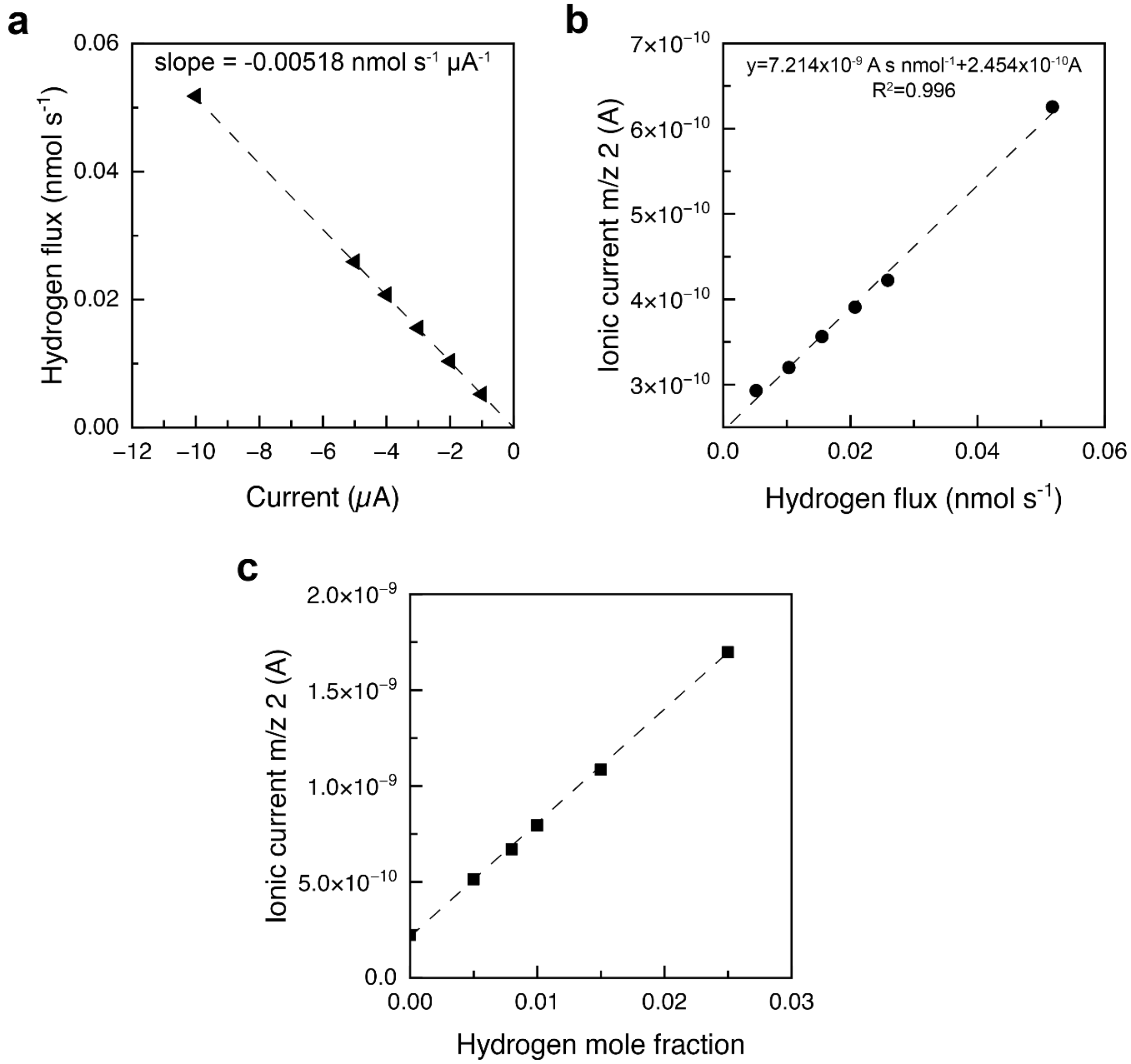

Supplementary Fig. 13. EC-MS H_2 calibration data. **(a)** Hydrogen evolution reaction EC-MS internal calibration data. Assuming 100% faradaic efficiency to produce hydrogen, the hydrogen flux was calculated as a function of the applied current. **(b)** EC-MS ionic current m/z 2 response to the introduction of hydrogen to the EC-MS system via the hydrogen evolution reaction. Linear fit of the data is shown as a dashed line and fitting parameters are shown. Data fit was used in Supplementary Eq. 1. **(c)** EC-MS H_2 external calibration data. Ionic current for m/z 2 in response to the introduction of H_2 to the EC-MS system. Hydrogen mole fraction and ionic current for m/z 2 were used in Supplementary Eq. 1.

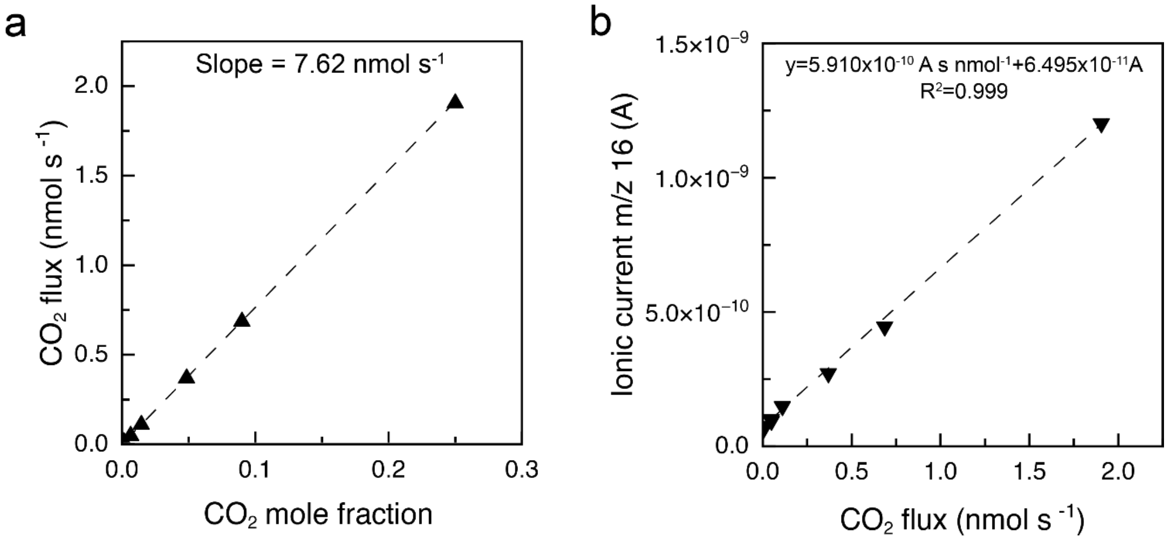

Supplementary Fig. 14. EC-MS CO_2 calibration data. **(a)** Calculation of CO_2 flux from the mole fraction of CO_2 introduced to the EC-MS system. The conversion factor of 7.62 nmol s^{-1} was calculated from the hydrogen internal and external calibrations. **(b)** EC-MS CO_2 external calibration data. Ionic current for m/z 16 in response to the introduction of CO_2 to the EC-MS system. Value of slope is used to convert ionic current recorded during experiments to the CO_2 flux.

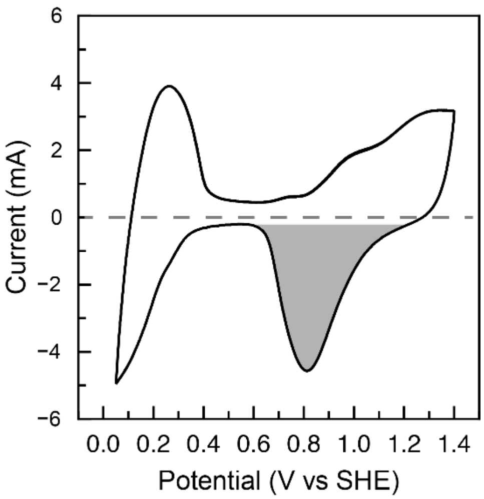

Supplementary Fig. 15. Representative post-experiment cyclic voltammogram used for surface area normalizing data. Performed in propane saturated 1 M $HClO_4$ at 60 °C in the electrochemical mass spectrometry experimental setup. Grey shaded area shows the integration of platinum oxide reduction charge ($C_{o\text{-}r}$). Scan rate: 50 mV s^{-1}. Here, a single measurement is plotted with $C_{o\text{-}r}$ = –0.022491 C. Cell resistance = 13.7±0.2 Ω. Data was not iR corrected.

EC-MS signals were collected for solutions containing varying concentrations of methanol, ethanol, and 2-propanol (Supplementary Fig. 16). The m/z 16 signal was, however, not correlated to the alcohol concentration and the m/z 16 signal is therefore not convoluted by the potential generation of oxygenates. A slight decrease in m/z 16 and 32 signals was observed as the concentration of alcohol was increased. We attribute this to the slow removal of O_2 from the EC-MS vacuum system with increased experiment time, as the concentration of alcohols was sequentially increased.

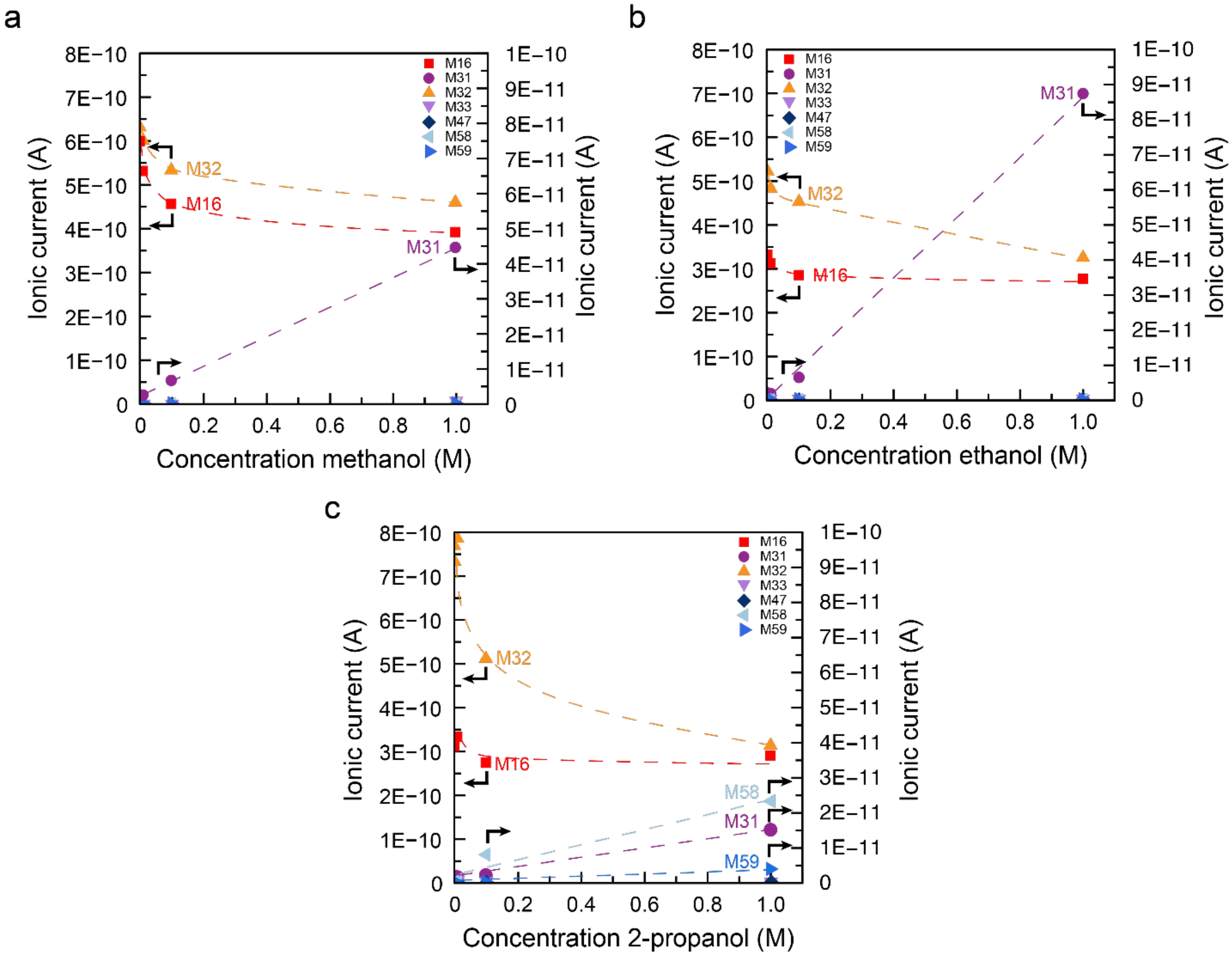

Supplementary Fig. 16. EC-MS detection of alcohols. "M" labels indicate m/z value. M16 and M32 are plotted using the left y-axis. M31, M33, M47, M58, M59 are plotted using the right y-axis. Dashed lines added as guides to the eye. A single measurement was made for each data point. Measurements were performed at room temperature. **(a)** Calibration for 0, 0.01, 0.1, and 1 M methanol. **(b)** Calibration for 0, 0.01, 0.1, and 1 M ethanol. **(c)** Calibration for 0, 0.001, 0.01, 0.1, and 1 M 2-propanol.

He Control Experiments

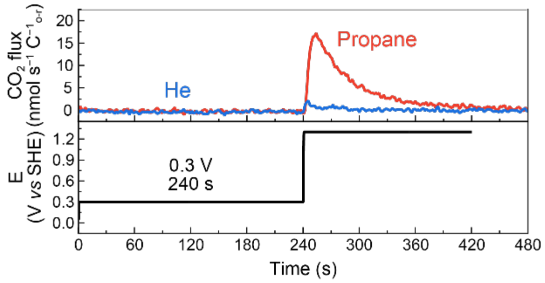

Supplementary Fig. 17. Representative electrode potential program and CO_2 flux observed during $E_{ads} = 0.3$ V, $t_{ads} = 240$ s adsorption experiments performed under He or propane. The CO_2 flux was calculated using the m/z 16 MS ionic current. Under He the CO_2 yield was 49.3 nmol C^{-1}_{o-r} CO_2 (Cell resistance = 17.0±1.6 Ω). Under propane the CO_2 yield was 884.7 nmol C^{-1}_{o-r} CO_2 (Cell resistance = 12.0±0.0 Ω). Each trace was from a single separate measurement.

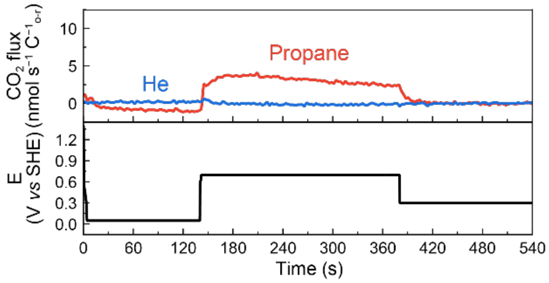

Supplementary Fig. 18. Representative electrode potential program and CO_2 flux observed during $E_{turnover} = 0.7$ V, $t_{turnover} = 240$ s constant-potential oxidation experiments performed under He or propane. The CO_2 flux was calculated using the m/z 16 MS ionic current. Under He the CO_2 yield was 20.2 nmol C^{-1}_{o-r} CO_2 (Cell resistance = 35.6 Ω). Under propane the CO_2 yield was 880.7 nmol C^{-1}_{o-r} CO_2 (Cell resistance = 35.8 Ω). Each trace was from a single separate measurement.

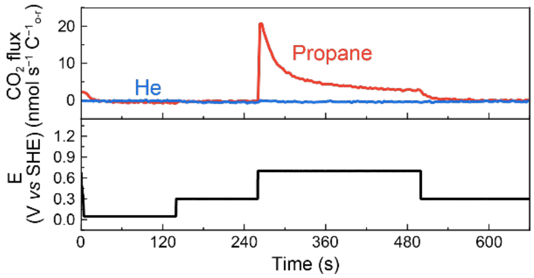

Supplementary Fig. 19. Representative electrode potential program and CO_2 flux during a conversion experiment using $E_{conv} = 0.7$ V and $t_{conv} = 240$ s, with pre-adsorption at $E_{ads} = 0.3$ V and $t_{ads} = 120$ s. The experiment was carried out under He or propane. The CO_2 flux was calculated using the m/z 16 MS ionic current. Under He the CO_2 yield was 19.0 nmol C^{-1}_{o-r} CO_2 (Cell resistance = 35.6 Ω). Under propane the CO_2 yield was 1406.9 nmol C^{-1}_{o-r} CO_2 (Cell resistance = 35.7 Ω). Each trace was from a single separate measurement.

Propane to He Gas Exchange Control Experiments

To determine if the presence or absence of propane during the oxidation step influences reaction outcomes, an experiment was performed where propane was adsorbed to the electrode at 0.3 V for 120 s before the gas supply was changed to He (Supplementary Fig. 20). We then maintained the potential at 0.3 V for 45 min under He before applying 1.3 V for 3 min to promote the oxidation of adsorbed propane. Upon changing the gas supply to He, a decrease of the major propane fragments (M28, M43, M44) was observed, which confirms the removal of solution phase propane (Supplementary Fig. 20a). Upon the application of 1.3 V, we observed peaks in M16 and M44 ionic currents, indicating the production of CO_2 from adsorbed propane (Supplementary Fig. 20b). Calculation of the CO_2 yield from the M16 peak area at 2960 s yielded 813 nmol $C^{-1}{}_{o\text{–}r}$ CO_2. This value was close to adsorption experiments performed at E_{ads} = 0.3 V for t_{ads} = 120 s under continuous propane flow (907 nmol $C^{-1}{}_{o\text{–}r}$ CO_2). The similar CO_2 yield (10% difference) suggests that propane does not significantly desorb from the surface after changing the gas supply to He and that the reaction outcome is not altered by the presence or absence of propane. No peak in the M32 signal was observed upon application of 1.3 V suggesting that no O_2 was produced and therefore does not contribute to the M16 signal. Furthermore, the absence of an increase in the M43 signal suggests that propane molecules were not being desorbed during the oxidation step.

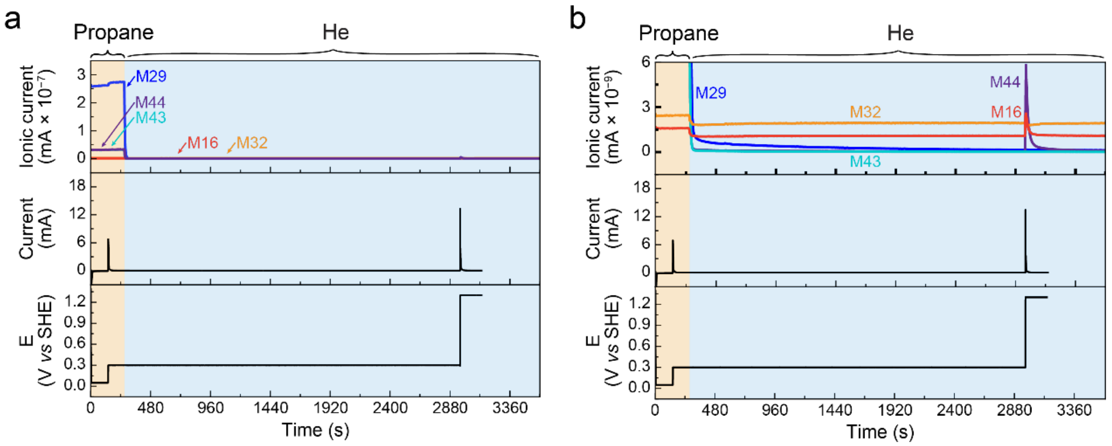

Supplementary Fig. 20. Modified adsorption experiment where after adsorption at E_{ads} = 0.3 V for t_{ads} = 120 s the gas supply was changed to He and the potential maintained at 0.3 V until stepping to 1.3 V at 2960 s. "M" notation indicate m/z value. Data is from a single measurement. **(a)** Larger ionic current scale showing the decay of the major propane fragments (M28, M43, M44) upon changing the gas supply to He **(b)** Magnified ionic current scale showing the M16 and M44 peak upon application of 1.3 V. (Cell resistance = 35.6 Ω)

Broad Spectrum Product Detection

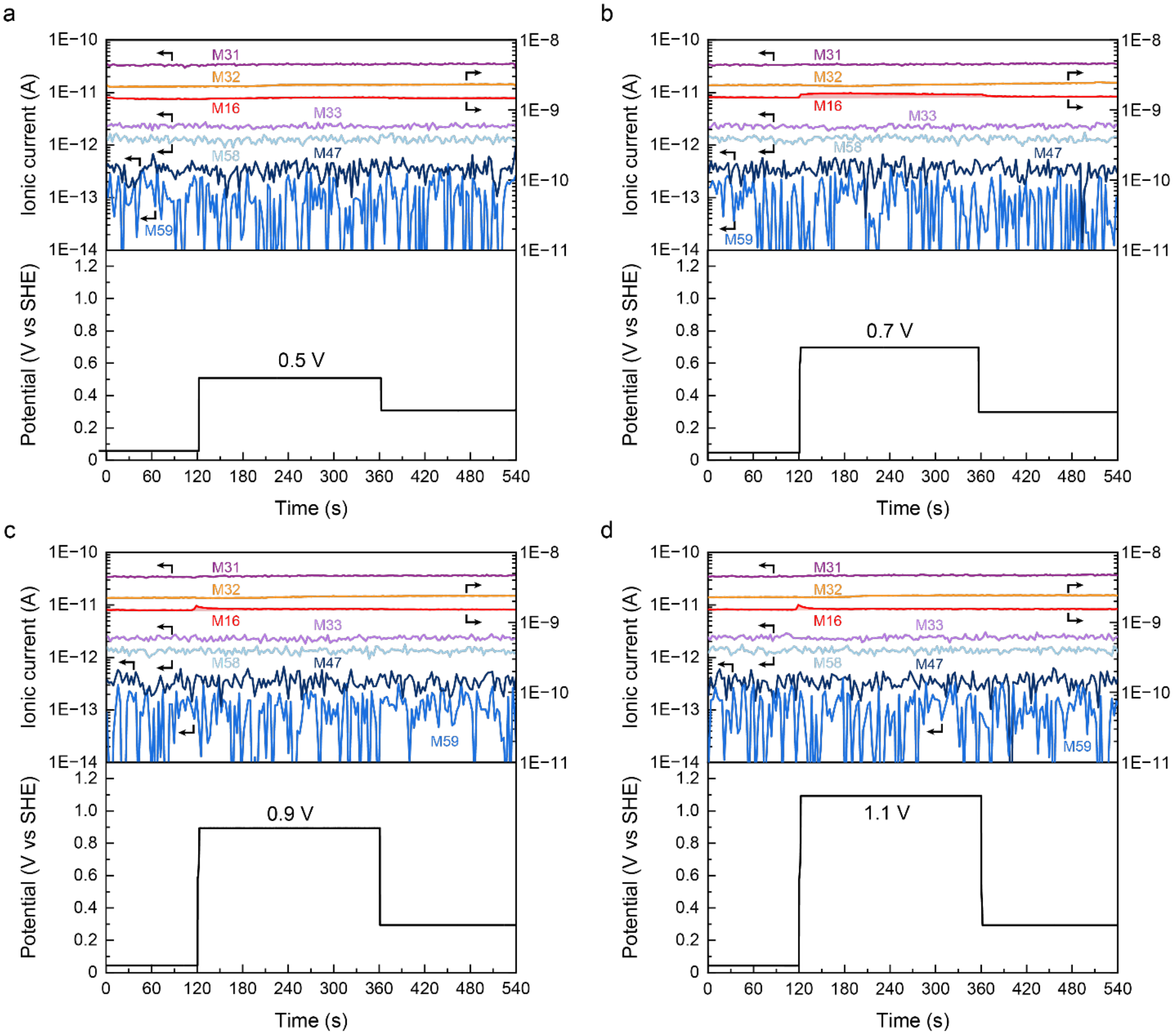

Supplementary Fig. 21. EC-MS ionic current signals during the constant-potential oxidation of propane-saturated solution at 60 °C. "M" notation indicate m/z value. Mass fragments associated with the generation of C1 (methanol, M31), C2 (ethanol, M47), and C3 (propanol, M59 and acetone, M58) oxygenates, O_2 (M32), and CO_2 (M16) that do not overlap with propane mass fragments are shown. Integrated areas under M16 curves are shown as shaded areas. Cell resistance = 35.6±0.1 Ω. A single measurement was performed for each constant-potential oxidation experiment. Applied potential: **(a)** 0.5 V, **(b)** 0.7 V, **(c)** 0.9 V, **(d)** 1.1 V.

During the application of each oxidative turnover potential, no increase in the ionic currents for the mass fragments related to oxygenate formation (m/z 31, 32, 33, 47, 58, 58) other than CO_2 (m/z 16) was observed. This is consistent with prior literature.[11,12]

Estimation of Diffusion-limited Adsorption Rate

The flux of a species via diffusion is given by:

$$J = -D\frac{dC}{dx}, \qquad \text{(S8)}$$

where J is the diffusion flux in nmol s^{-1} cm^{-2}, D is the diffusion coefficient, and $\frac{dC}{dx}$ is the concentration gradient. The diffusion coefficient, of propane at 60 °C in water was extrapolated to be 2.07×10^{-5} cm^2 s^{-1} from the diffusion measurements performed by Witherspoon and Saraf as shown in Supplementary Fig. 22.[13] The concentration gradient was calculated from the concentration difference between the bulk solution and electrode surface. Here we assume that under diffusion limited adsorption, the concentration at the electrode surface is zero. The concentration of propane in the bulk was approximated to be 2.0×10^{-3} M based on measurements reported by Morrison and Billett.[14] The thickness of the diffusion layer was assumed to be 100 μm which is the thickness of the EC-MS cell electrolyte compartment (the lowest possible flux scenario for this cell and these conditions), and a linear concentration profile was assumed. Substitution into Supplementary Eq. 8 and solving for J yields a flux of 4.1 nmol propane s^{-1} cm^{-2}. Considering that propane adsorption at 0.4 V for 240 s closely approaches saturation (Fig. 4b) and yielded approximately 28 nmol CO_2, we can estimate that 9.3 nmol propane was adsorbed to the 0.196 cm^2_{geo} electrode. This corresponds to a coverage of 47.4 nmol propane cm^2_{geo}. Diffusion limited adsorption of propane should therefore reach full saturation in approximately 11.5 s. As shown in Figs. 2b and 4b, full saturation was not achieved within 11.5 s thus indicating that the adsorption of propane was not limited by propane diffusion.

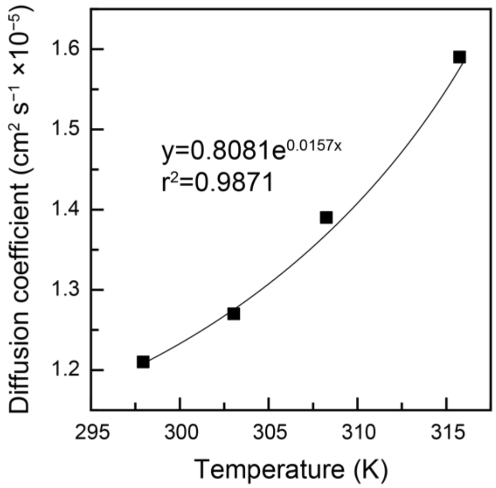

Supplementary Fig. 22. Diffusion coefficient of propane in water as a function of temperature as reported by Witherspoon and Saraf.[13] Data was fit with an exponential growth function in Microsoft Excel based on the assumption of an Arrhenius-type relationship. The diffusion coefficient of propane in water at 60 °C was calculated from the fit.

Additional Data – Cyclic Voltammetric Study of Propane Oxidation at 80 °C

Cyclic voltammetry experiments were performed in a glass 5-neck flask. All experiments were performed using a Gamry 1010 potentiostat controlled using Gamry Framework (Version 7.10.3) with a three-electrode configuration. The platinized Pt catalyst was mounted in a Pine Research E5TQ Rotating Disk Electrode ChangeDisk. The Rotating Disk Electrode ChangeDisk was inserted into a Pine Research MSR Rotator. 1 M perchloric acid was used as the electrolyte for all experiments. The reaction vessel was heated to 80 ± 1 °C for all experiments using an oil bath and Corning (PC-420D + thermocouple) hot plate. In the 5-neck flask electrochemical cell, 40 mL of 1 M perchloric acid was sparged with Ar at a flow rate of 100 sccm for 30 min while the solution was stirred at 400 rpm with a stir bar to remove O_2 before each experiment. Gas flow was controlled using Alicat Mass Flow Controllers. Ar background experiments were completed first using an Ar flow rate of 50 sccm into the reactor headspace. During Ar experiments, the reactor headspace was purged with 50 sccm Ar gas and no stirring was performed. After Ar experiments, the solution was sparged with propane at 50 sccm for 30 min while the solution was stirred at 400 rpm with a stir bar. During propane experiments, the reactor headspace was purged with 50 sccm propane gas and no stirring was performed. An Ag/AgCl (3.0 M KCl, BASi RE-5B) reference electrode was interfaced with the solution via a PTFE capillary and glass fritted double junction containing approximately 10 mL of 1 M perchloric acid. A Pt mesh counter electrode (99.9%, 100 mesh, Sigma-Aldrich) with a Ti wire (99.7%, Sigma-Aldrich) current collector was interfaced with the reaction solution via a glass compartment separated by a porous glass frit containing approximately 5 mL of 1 M perchloric acid. A Pine Research Gas-Purged Bearing Assembly was used to seal the electrochemical cell and 20 sccm Ar was delivered to the Gas-Purged Bearing Assembly at all times. Potentiostatic electrochemical impedence spectroscopy from 0.2 Hz to 0.1 MHz was used to measure a cell resistance of 2.3 Ω. Data was not iR corrected.

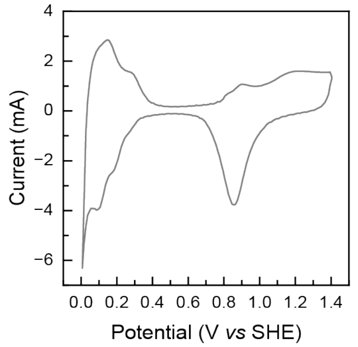

Supplementary Fig. 23. Cyclic voltammogram of platinized platinum electrode in Ar saturated 1 M $HClO_4$ at 80 °C from 0 – 1.4 V vs SHE. Scan rate was 50 mV s^{-1}. Data was smoothed in OriginLab 2022b using a 20 mV window Adjacent-Averaging. Experimental set-up as described in the Cyclic Voltammetric Study of Propane Oxidation at 80 °C section. cell resistance = 2.3 Ω. Data was not iR corrected.

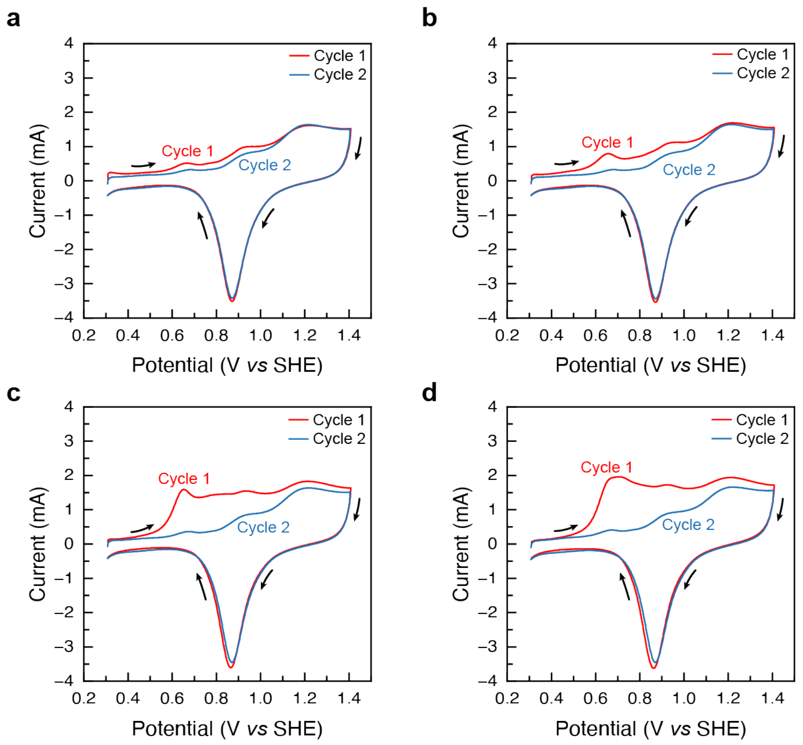

Supplementary Fig. 24. Cyclic voltammograms following propane adsorption at 0.3 V for t_{ads} = 10, 60, 300, and 900 s. Scan rate was 50 mV s^{-1}. Experiments set-up as described in Cyclic Voltammetric Study of Propane Oxidation at 80 °C. Cell resistance = 2.3 Ω. Data was not iR corrected. **(a)** t_{ads} = 10 s. **(b)** t_{ads} = 60 s. **(c)** t_{ads} = 300 s. **(d)** t_{ads} = 900 s.

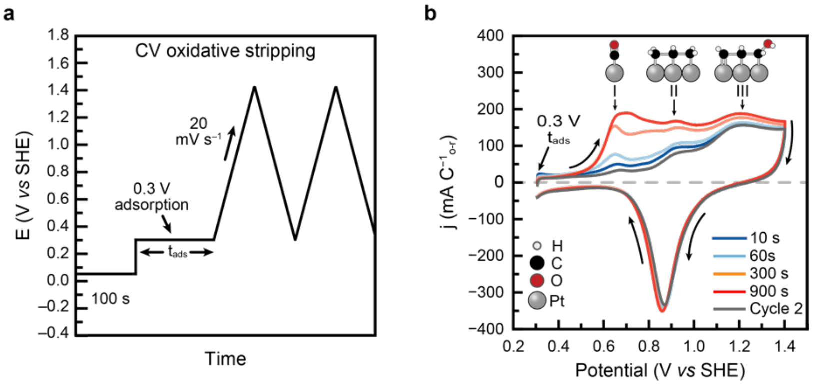

Supplementary Fig. 25. Electrode potential program and overlayed results for Cyclic Voltammetric Study of Propane Oxidation at 80 °C experiments. **(a)** Electrode potential program for the study of propane adsorption at 0.3 V for t_{ads} = 10, 60, 300, and 900 s and subsequent cyclic voltammetric oxidative stripping. **(b)** Cyclic voltammetric oxidative stripping traces after adsorption of propane at 0.3 V for 10, 60, 300, and 900 s. Cycle 2 immediately follows Cycle 1 and represents the case for t_{ads} = 0 s. A single Cycle 2 trace (from t_{ads} = 10 s) is shown for clarity. Oxidative peaks are labeled Peak I, II, and III where each Peak correspond to the oxidation of the indicated adsorbate. Only one of several possible multi-carbon adsorbates corresponding to Peaks II and III are shown for simplicity. Scan rate = 20 mV s^{-1}. Cell resistance = 2.3 Ω. Data was not iR corrected. Data was smoothed with the Savitzky-Golay method with 5 mV window and second order polynomial in Origin Lab (Version 2022b).

Photographs of Experimental Setups
I. EC-MS Setup

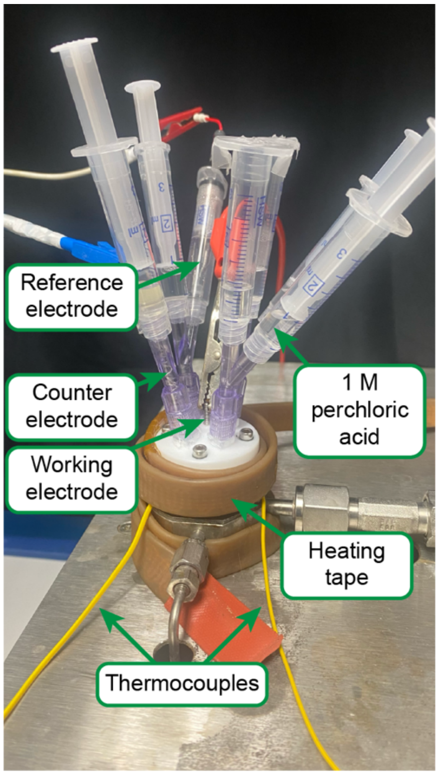

Supplementary Fig. 26. EC-MS experimental set up. Electrochemical cell components and electrodes are installed. Heating tape is installed around the EC-MS electrochemical cell mounting block and the electrochemical cell.

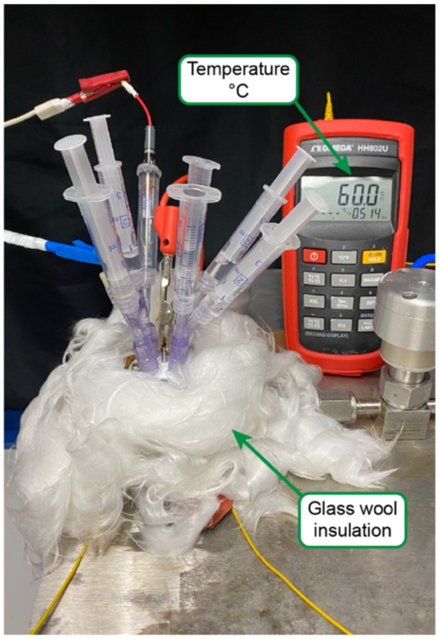

Supplementary Fig. 27. EC-MS experimental set up. Electrochemical cell is heated to 60 °C and insulated with glass wool to improve temperature stability. All EC-MS experiments performed in this configuration.

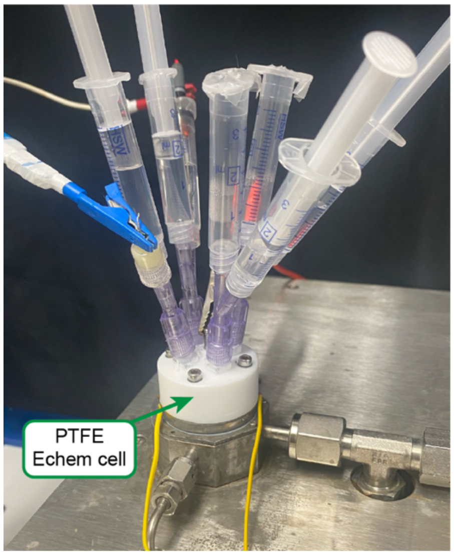

Supplementary Fig. 28. EC-MS experimental set up. Shown without heating tape to improve visibility of the electrochemical cell and EC-MS mounting block in the photograph.

II. Rotating Disc Electrode Setup

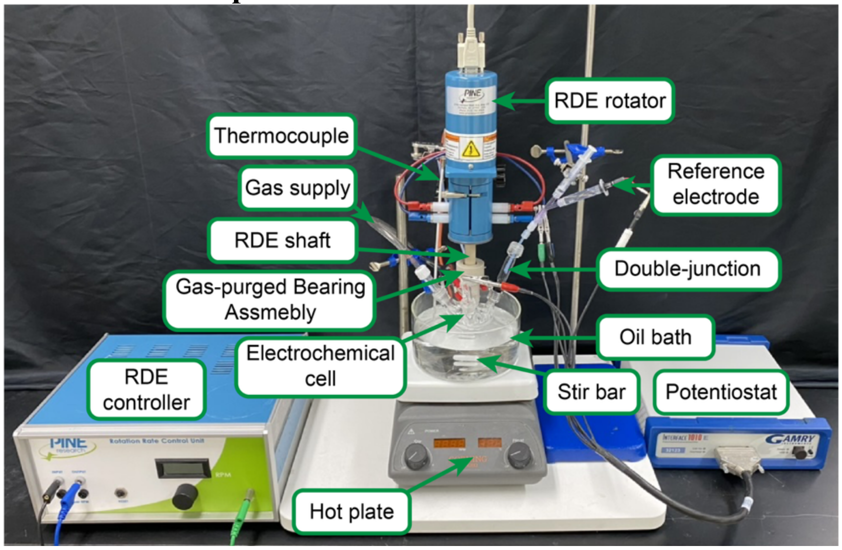

Supplementary Fig. 29. Photograph of Rotating Disk electrode set-up used during Cyclic Voltammetric Study of Propane Oxidation at 80 °C experiments.

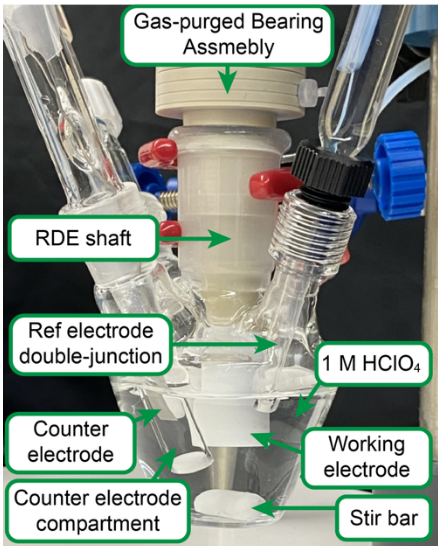

Supplementary Fig. 30. Photograph of Rotating Disk electrode electrochemical cell set-up used during Cyclic Voltammetric Study of Propane Oxidation at 80 °C experiments.

Catalyst Characterization

I. Catalyst Preparation for Material Characterization

Platinized Pt electrodes were prepared as described in the *Catalyst Preparation* section. After catalyst deposition and rinsing with Milli-Q water, the electrode was mounted in the EC-MS cell. In a 1 M $HClO_4$ solution at room temperature, 20 CV cycles from 0 to +1.4 V were performed at 50 mV s^{-1} to pre-condition the electrode. The electrode was then removed from the cell and rinsed with Milli-Q water. The sample was then allowed to dry under ambient conditions before being placed in a PTFE sample holder for transport to analysis facilities.

II. X-ray Diffraction

X-ray Diffraction (XRD) was performed on the platinized Pt catalyst using a Bruker D8 Discovery X-ray diffractometer using a Cu K_α X-ray source, 0.5 mm spot size, and a Vantec 500 area detector. The X-ray diffraction pattern is shown in Supplementary Fig. 31 and agrees with the XRD pattern of Pt in the PDF-5+ 2025 database.[15]

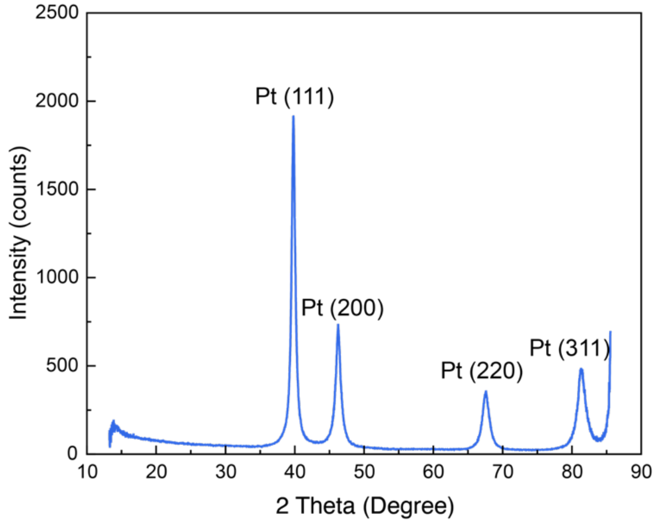

Supplementary Fig. 31. X-ray diffraction pattern of platinized Pt catalyst. Peaks have been labeled with corresponding reflections.

Reflections were assigned according to those listed for Pt in the PDF-5+ 2025 database. Crystallite size was determined using Scherrer analysis available in the Bruker DIFFRAC.EVA software using a shape factor, K, equal to 0.89 and instrument line broadening of 0.05. The mean crystallite size was determined to be 136.8 Å when analyzing the peak broadening of the $2\theta = 39.810$ peak.

III. X-ray Photoelectron Spectroscopy

Surface-sensitive elemental analysis was performed on the platinized Pt catalyst using X-ray photoelectron spectroscopy (XPS). A Thermo k-alpha X-ray photoelectron spectrometer with an Al K_α X-ray source with a hemispherical electron energy analyzer was used. The results are shown in Supplementary Fig. 32. The peak binding energy positions were compared to reference materials.[16–18]

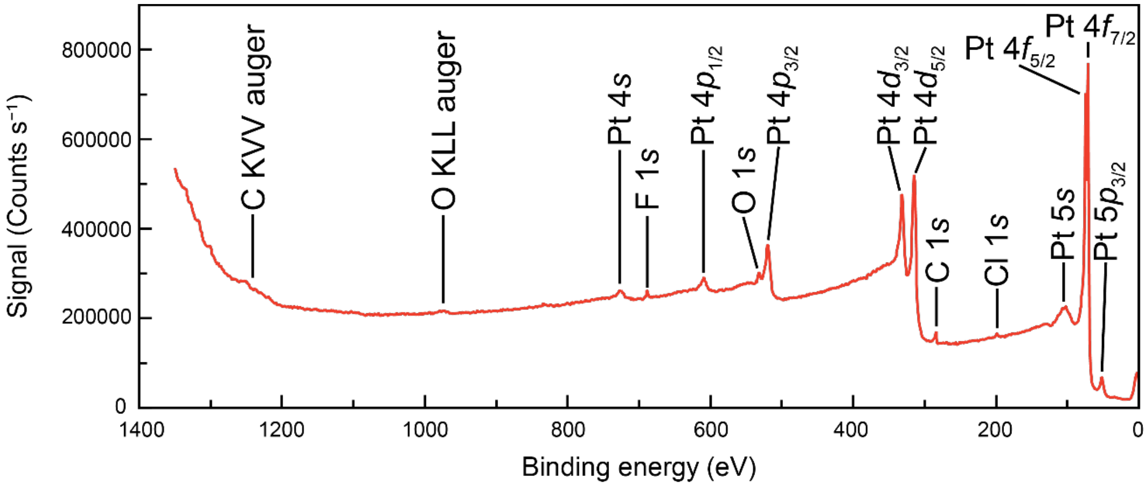

Supplementary Fig. 32. X-ray photoelectron spectroscopy spectra of platinized Pt catalyst. The origin of each peak is labeled.

The peak at 71.1 eV corresponds closely to the reference value of 71.2 eV for Pt $4f_{7/2}$. This peak is not shifted to higher binding energies, indicating an absence of Pt binding to electronegative elements such as O, thus suggesting that Pt exists predominantly in the reduced state. If Pt oxides or Pt hydroxides were present, a Pt $4f_{7/2}$ binding energy between 74 – 75 eV or 72 – 73 eV would likely be observed, respectively.[16]

The presence of trace elements was analyzed using high-resolution XPS spectra (Supplementary Fig. 33). A Cl 1*s* binding energy of 198.1 eV was observed. This binding energy is closest to the Cl 1*s* binding energy found for tetrachloroplatinate (198.4 – 198.7 eV) and hexachloroplatinate (198.9 eV) samples. This suggests that the observed trace Cl likely exists in a complexed form, likely residues from the chloroplatinic acid deposition solution. The detected Cl does not come from the perchloric acid electrolyte since a Cl 1*s* binding energy of 208.33 eV is expected for perchlorates.[16] The dominant observed C and O peaks likely correspond to adventitious carbon and water, which are nearly unavoidable and therefore common in XPS analysis. A small peak at 688.7 eV might point to C–F bonding, potentially originating from the PTFE sample holder used to transport and handle the sample. No presence of Pb, which is used as a promotor during electrodeposition, was detected.

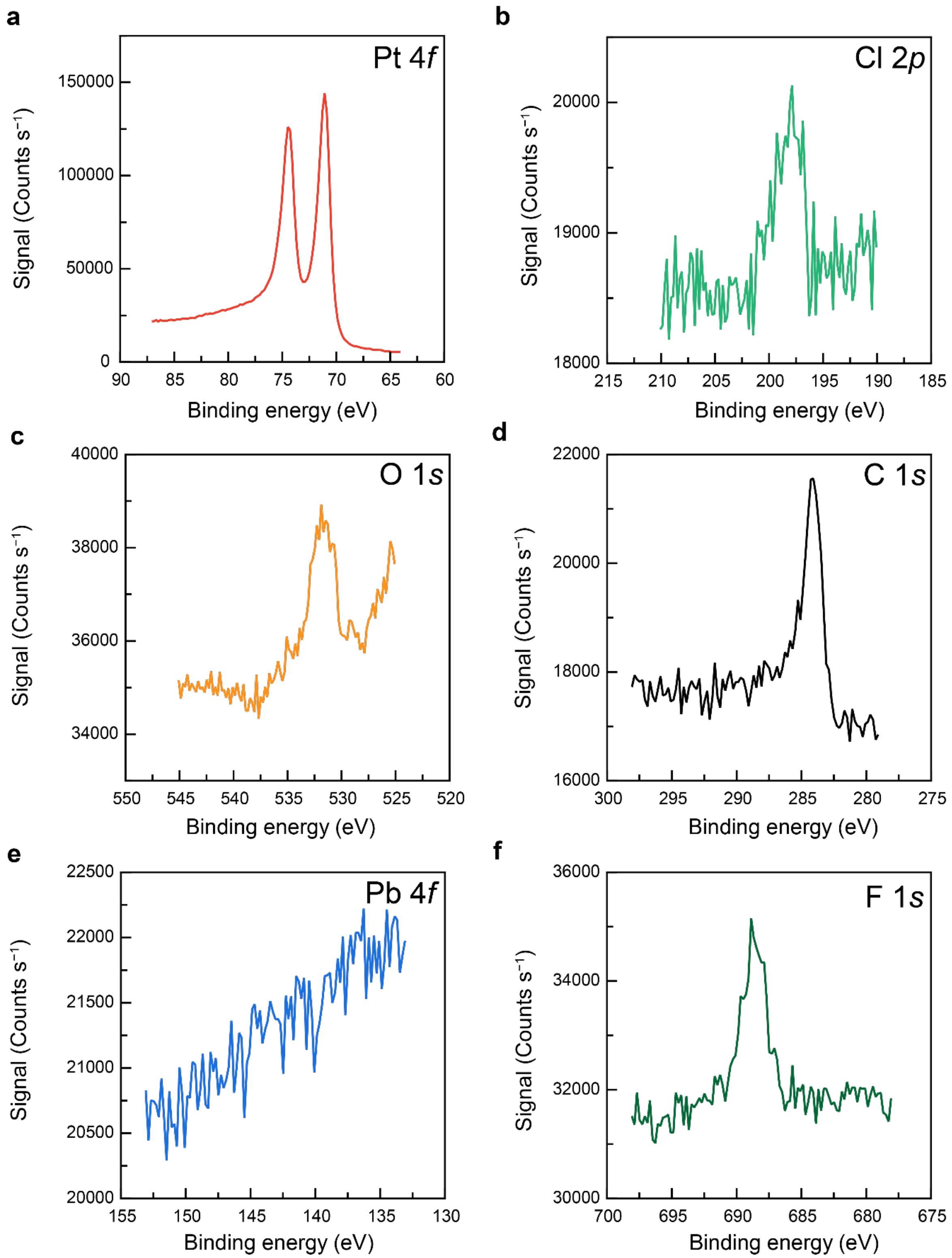

Supplementary Fig. 33. High-resolution x-ray photoelectron spectroscopy of platinized Pt catalyst. Ten scans were collected for each spectrum. **(a)** Pt 4*f*. **(b)** Cl 2*p*. **(c)** O 1*s*. **(d)** C 1*s*. **(e)** Pb 4*f*. **(f)** F 1*s*.

IV. Scanning Electron Microscopy

Scanning electron microscopy (SEM) was used to assess the topography of the platinized Pt catalyst. SEM analysis was performed using a Zeiss Gemini 450 at an acceleration voltage of 20.00 kV. We observed that the platinized Pt catalyst was composed of hierarchical, fractal-like morphology.

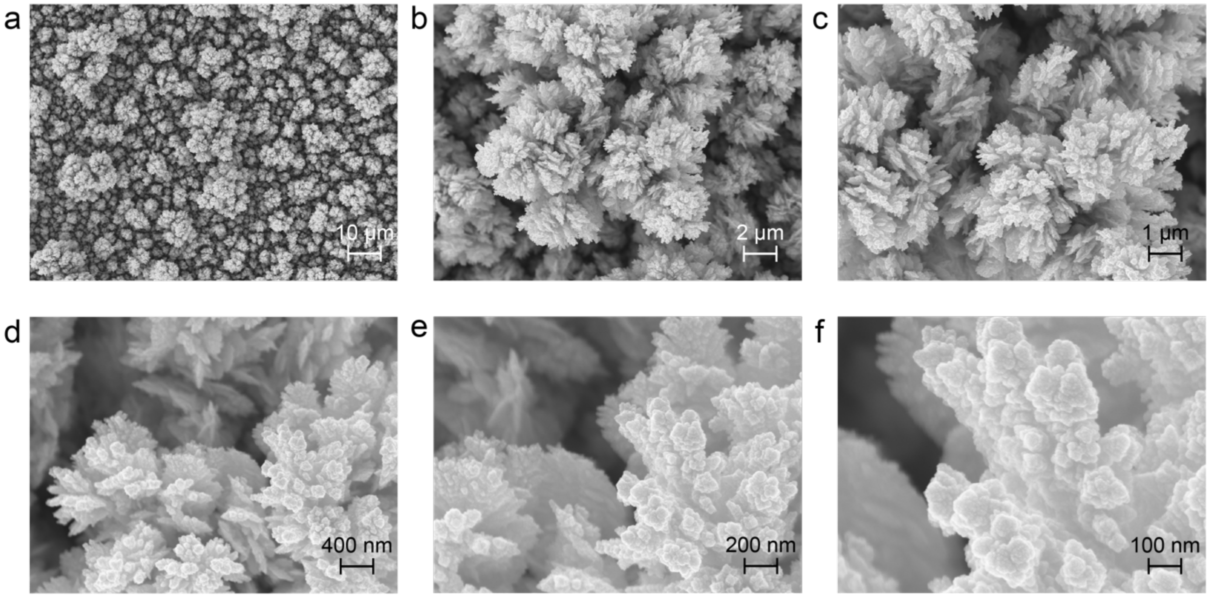

Supplementary Fig. 34. Scanning electron microscope images of the platinized platinum catalyst. Magnification: **(a)** 1 k, **(b)** 5 k, **(c)** 10 k, **(d)** 25 k, **(e)** 50 k, **(f)** 100 k.

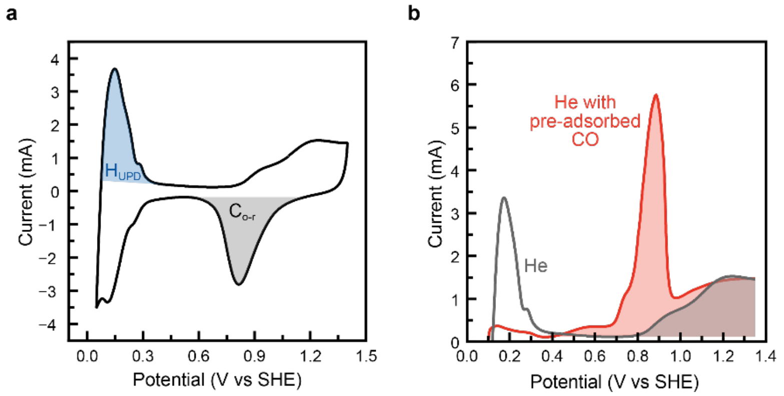

Supplementary Fig. 35. Voltammetric measurements for the determination of electrochemical surface area (ECSA). **(a)** Cyclic voltammogram from 0.05 to 1.4 V performed at a scan rate of 20 mV s^{-1}. Blue shading indicates the integrated area associated with the charge passed for the desorption of hydrogen in the hydrogen underpotential deposition (H_{UPD}) region. Grey shading indicates the integrated area associated with the charge passed for platinum oxide reduction (C_{o-r}). **(b)** Linear sweep voltammograms (LSVs) from 0.1 to 1.35 V performed at scan rates of 20 mV s^{-1}. The red LSV was performed in He saturated 1 M $HClO_4$ after adsorbing CO to the electrode for 20 min at 0.1 V in CO saturated 1 M $HClO_4$. The red shading indicates the integrated area associated with the oxidation of pre-adsorbed CO. The grey LSV was performed in He saturated 1 M $HClO_4$ with no chronoamperometric hold at 0.1 V. The grey shaded area indicates the integrated area associated with surface oxide formation. Measurements performed using the EC-MS set-up at room temperature. Cell resistance = 10.8 ±0.1 Ω. Data was not iR corrected.

EC-MS Electrochemical Cell Configuration

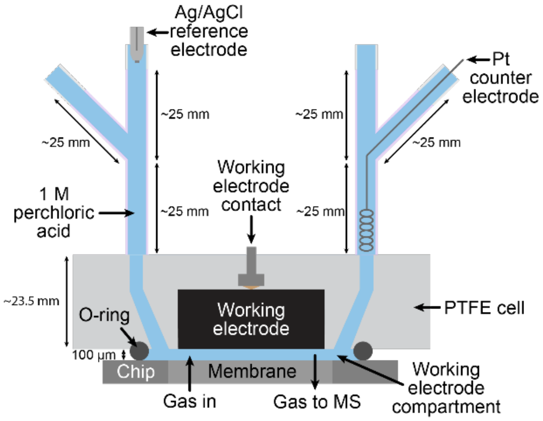

Supplementary Fig. 36. Diagram of electrochemical cell and interface with mass spectrometer inlet chip. Image is not to scale.

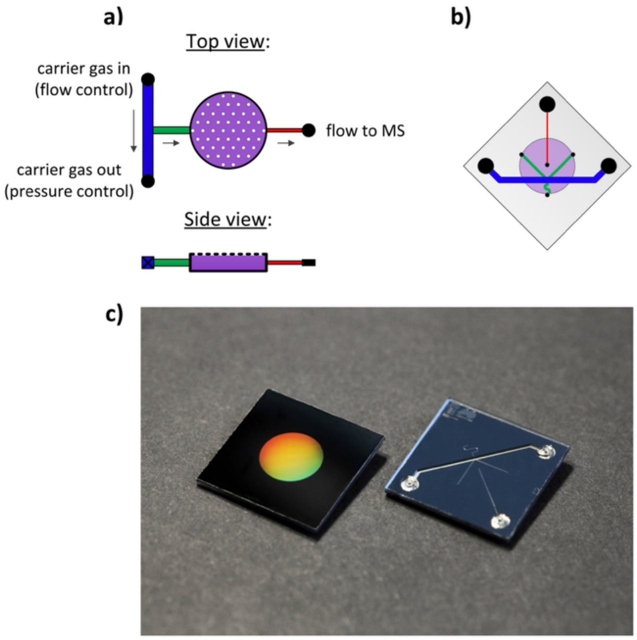

Supplementary Fig. 37. EC-MS membrane chip. **(a)** Diagram of gas flow channels within the membrane chip. The blue area indicates the carrier gas flow channel, the green lines indicate the delivery channel to the sampling volume, the purple area indicates the sampling volume, the red line indicates the capillary inlet channel to the mass spectrometer, and black areas indicate the points of connection between the membrane chip and the system. **(b)** Diagram of membrane chip. **(c)** Photograph of the front side of the membrane chip showing the membrane surface (left). Photograph of the back side of the membrane chip showing the gas flow channels (right). Reprinted from Electrochimica Acta, 268, Trimarco, D.B., Scott, S.B., Thilsted, A.H., Pan, J.Y., Pedersen, T., Hansen, O., Chorkendorff, I., Vesborg, P.C.K., Enabling real-time detection of electrochemical desorption phenomena with sub-monolayer sensitivity, 520–530, Copyright (2018), with permission from Elsevier.

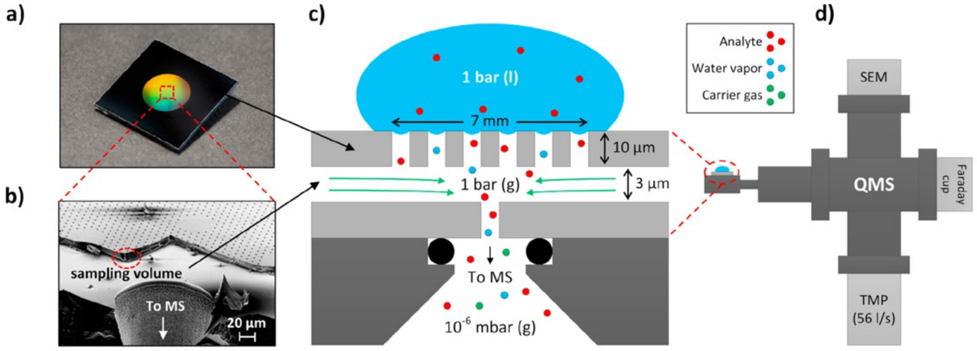

Supplementary Fig. 38. Images of the membrane chip and illustrations of EC-MS function. **(a)** Photograph of front face of membrane chip. **(b)** Cross-section Scanning Electron Microscopy image of membrane chip. The sampling volume is circled in red. **(c)** Cross-section illustration of analyte (red), water vapor (blue), and carrier gas (green) flow within the membrane chip (light grey). Water and analyte enter the system through the chip membrane. Carrier gas is delivered through the chip. Water, analyte, and carrier gas enter the mass spectrometer (MS) for analysis. Stainless steel membrane chip mounting surface is shown in dark grey and O-ring gaskets are shown in black. **(d)** Membrane chip inlet in relation to the mass spectrometer system. Reprinted from Electrochimica Acta, 268, Trimarco, D.B., Scott, S.B., Thilsted, A.H., Pan, J.Y., Pedersen, T., Hansen, O., Chorkendorff, I., Vesborg, P.C.K., Enabling real-time detection of electrochemical desorption phenomena with sub-monolayer sensitivity, 520–530, Copyright (2018), with permission from Elsevier.

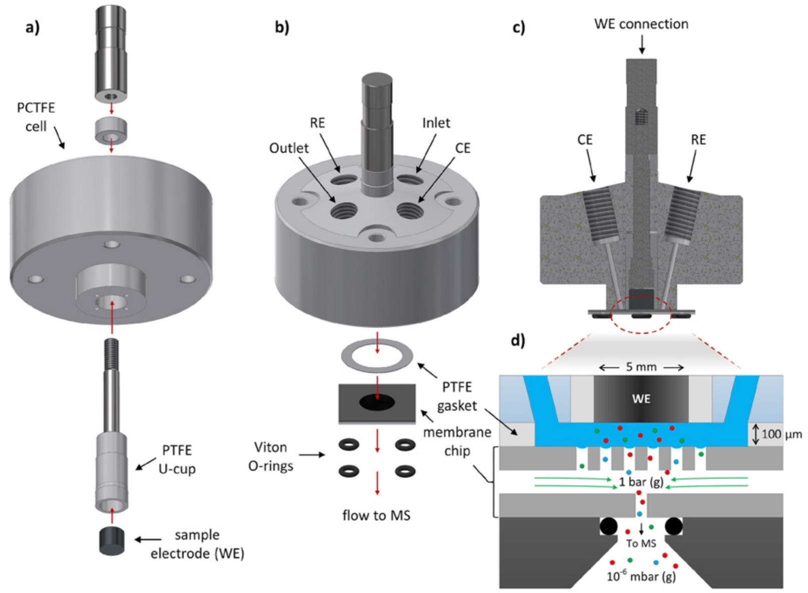

Supplementary Fig. 39. Diagram of electrochemical cell, working electrode, and membrane chip inlet. **(a)** Schematic diagram of stagnant thin-layer electrochemical and electrode mounting hardware. **(b)** Diagram showing the top view of the electrochemical cell. The mounting order of the gaskets and O-rings used to interface the electrochemical cell with the membrane chip and mass spectrometer system are shown. **(c)** Cross-section diagram of the electrochemical cell. **(d)** Cross-section diagram of the interface between the electrochemical cell, membrane inlet chip, and mass spectrometer system. Reprinted from Electrochimica Acta, 268, Trimarco, D.B., Scott, S.B., Thilsted, A.H., Pan, J.Y., Pedersen, T., Hansen, O., Chorkendorff, I., Vesborg, P.C.K., Enabling real-time detection of electrochemical desorption phenomena with sub-monolayer sensitivity, 520–530, Copyright (2018), with permission from Elsevier.

References

1. Feltham, A. M. & Spiro, M. Platinized platinum electrodes. *Chem Rev* **71**, 177–193 (1971).
2. Lucky, C., Jiang, S., Shih, C.-R., Zavala, V. M. & Schreier, M. Understanding the interplay between electrocatalytic C(sp3)–C(sp3) fragmentation and oxygenation reactions. *Nat Catal* **7**, 1021–1031 (2024).
3. Grdeń, M., Łukaszewski, M., Jerkiewicz, G. & Czerwiński, A. Electrochemical behaviour of palladium electrode: Oxidation, electrodissolution and ionic adsorption. *Electrochim Acta* **53**, 7583–7598 (2008).
4. Boyd, M. J. *et al.* Electro-Oxidation of Methane on Platinum under Ambient Conditions. *ACS Catal* **9**, 7578–7587 (2019).
5. Trimarco, D. B. *et al.* Enabling real-time detection of electrochemical desorption phenomena with sub-monolayer sensitivity. *Electrochim Acta* **268**, 520–530 (2018).
6. Trimarco, D. B., Pedersen, T., Hansen, O., Chorkendorff, I. & Vesborg, P. C. K. Fast and sensitive method for detecting volatile species in liquids. *Review of Scientific Instruments* **86**, (2015).
7. Vesborg, P. C. K. *et al.* Quantitative measurements of photocatalytic CO-oxidation as a function of light intensity and wavelength over TiO2 nanotube thin films in μ-reactors. *Journal of Physical Chemistry C* **114**, 11162–11168 (2010).
8. Cairns, E. J. & Breitenstein, A. M. The Kinetics of Propane Adsorption on Platinum in Hydrofluoric Acid. *J Electrochem Soc* **114**, 764–772 (1967).
9. Cairns, E. J., Breitenstein, A. M. & Scarpellino, A. J. The Kinetics of Adsorption, Surface Reaction, and Electrochemical Oxidation of Propane on Platinum in Hydrofluoric Acid. *J Electrochem Soc* **115**, 569 (1968).
10. Gilman, S. Studies of hydrocarbon surface processes by the multipulse potentiodynamic method. Part 1.—Kinetics and mechanisms of ethane adsorption on platinum. *Trans. Faraday Soc.* **61**, 2546–2560 (1965).
11. Binder, H., Köhling, A., Krupp, H., Richter, K. & Sandstede, G. Electrochemical Oxidation of Certain Hydrocarbons and Carbon Monoxide in Dilute Sulfuric Acid. *J Electrochem Soc* **112**, 355 (1965).
12. Bruckenstein, S. & Comeau, J. Electrochemical mass spectrometry. Part 1.—Preliminary studies of propane oxidation on platinum. *Faraday Discuss. Chem. Soc.* **56**, 285–292 (1973).
13. Witherspoon, P. A. & Saraf, D. N. Diffusion of Methane, Ethane, Propane, and n-Butane in Water from 25 to 43°. *J Phys Chem* **69**, 3752–3755 (1965).
14. Morrison, T. J. & Billett, F. The salting-out of non-electrolytes. Part II. The effect of variation in non-electrolyte. *J Chem Soc* 3819 (1952).
15. PDF-5+ 2025 database. https://pdf5web.icdd.com/2025/index.htm.
16. Moulder, J. F., Stickle, W. F., Sobol, P. E. & Bomben, K. D. *Handbook of X-Ray Photoelectron Spectroscopy*. vol. 1 (Perkin-Elmer Corporation, Eden Prairie, 1992).
17. ThermoFisher Scientific Inc. Table of Elements: X-ray photoelectron spectroscopy of atomic elements. https://www.thermofisher.com/us/en/home/materials-science/learning-center/periodic-table.html.
18. National Institute of Standards and Technology. NIST X-ray Photoelectron Spectroscopy Database (SRD 20), Version 5.0. https://srdata.nist.gov/xps/ChemicalName.
